# Barriers to accessing newer antibiotics in countries with high burden of bacterial antimicrobial resistant infections—a qualitative study

**DOI:** 10.1016/j.lanwpc.2026.101917

**Published:** 2026-07-16

**Authors:** Shweta R. Singh, Alejandro Blanco-Arévalo, Atharvi Gupta, Jia Yi Chee, Alec Ann Alissa F. Aligui, Stanton Winter Hor, Yiyun Shou, Rathi Saravanan, James Leong, Esmita Charani, Yin Mo

**Affiliations:** a ADVANCE-ID, Saw Swee Hock School of Public Health, National University of Singapore, Singapore; b Centro de Investigación Biomédica en Red de Enfermedades Infecciosas (CIBERINFEC), Instituto de Salud Carlos IlI, Madrid, Spain; c Infectious Diseases Department, Bellvitge University Hospital, L’Hospitalet de Llobregat, Barcelona, Spain; d Clinical and Translational Research Institute, The Medical City, Ortigas Avenue, Pasig City, Philippines; e Clinton Health Access Initiative (CHAI), Boston, MA, United States; f Lloyd’s Register Foundation Institute for the Public Understanding of Risk, National University of Singapore, Singapore; g School of Medicine and Psychology, The Australian National University, Canberra, Australia; h Centre of Regulatory Excellence (CoRE), Duke-NUS Medical School Level 6, 8 College Road, 169857, Singapore; i Division of Infectious Diseases and HIV Medicine, Department of Medicine, University of Cape Town, Cape Town, South Africa; j Faculty of Health and Life Sciences, University of Liverpool, Liverpool, United Kingdom; k Infectious Diseases Translational Research Programme, Yong Loo Lin School of Medicine, National University of Singapore, Singapore; l Mahidol-Oxford Research Unit, Mahidol University, Bangkok, Thailand; m Center for Tropical Medicine and Global Health, Nuffield Department of Medicine, University of Oxford, Oxford, UK

**Keywords:** AMR, Antibiotic access, Equitable access, Stakeholder interviews

## Abstract

**Background:**

Bacterial antimicrobial resistant (AMR) infections pose a significant health threat. Newer antibiotics designed to treat resistant pathogens may offer superior efficacy and fewer side effects compared to older alternatives. However, access to newer antibiotics remains limited in high AMR burden Asian countries. We aimed to explore stakeholder perspectives on barriers and potential solutions to improving access to newer antibiotics across Asia.

**Methods:**

We conducted a qualitative study using semi-structured interviews with stakeholders across Asia between April 2024 and April 2025. Stakeholder groups included in-depth, multi-stakeholder interviews with Asian policymakers, drug regulators, healthcare professionals, pharmaceutical companies, global health actors, health technology assessors, and patient advocacy groups. We employed maximum variation sampling to understand participants’ unique perspectives on antibiotic access challenges. Interviews were audio-recorded, transcribed verbatim, and analysed using a seven-stage framework analysis.

**Findings:**

We interviewed 40 of 79 (50.6%) invited participants from Cambodia, China, India, Indonesia, Japan, Malaysia, Singapore, Sri Lanka and Thailand. Findings revealed a cascading set of barriers which highlighted deeper structural and political fractures in regional health governance. Firstly, misaligned mindsets were shaped by differing perceptions of AMR urgency between high and low-income countries causing fragmented implementation of solution strategies. These divergent perspectives influenced disjointed and short-term interventions, including reliance on inefficient workaround access mechanisms, and lack of sustained antibiotic stewardship and infection prevention and control policies. Third, systemic underinvestment in infrastructure, clinical trial capacity, regulatory processes, and market-shaping mechanisms further constrained access. Finally, in the absence of coordinated leadership, responsibilities remained siloed across stakeholders, with global health actors unable to fill the governance and accountability gap.

**Interpretation:**

Equitable access to newer antibiotics relies on commitments to strengthening health systems’ infrastructure and facilitating coherent evidence generation. Improved coordination among key stakeholders including government-private-global health actor partnerships, regulatory harmonisation and pooled procurement could significantly alleviate immediate challenges.

**Funding:**

This work was supported by the 10.13039/501100000286British Academy, 10.13039/501100001349National Medical Research Council (NMRC) Singapore and The Good Clinical Trials Collaborative (GCTC).

**Research in context**

**Evidence before this study**

We conducted a comprehensive search across Pubmed, EMBASE, CINAHL, Google Scholar, UpToDate, Clinical Key, major newspapers, policy documents, position papers, and official reports to identify publications examining factors affecting equitable access to FDA-approved antibiotics. Search was conducted from 5 February 2024 to 25 October 2024; terms included “access,” “novel”, “new”, “antibiotics”, “AMR”, “challenges”, “Asia”, “healthcare”, “pharmaceutical,” “regulatory,” “Ministry of Health”, “Health Technology Assessment”, “healthcare providers”, and “policy”. The search focused on literature published between January 2010 to October 2024 and yielded 134 relevant publications. These studies described challenges across seven stakeholder groups: policy makers, regulators, healthcare professionals, industry experts, global health actors (which included major international organisations influencing antibiotic access and antimicrobial resistance policy through funding, research, technical guidance, or coordination), health technology assessors, patients and patient advocates. While many antibiotic trials were conducted in high-burden regions such as Asia and Africa, post-approval access remained constrained for populations and health systems in these countries. Identified barriers included: limited and fragmented epidemiological data, inadequate health economic assessments, complex regulatory frameworks, manufacturing constraints, inefficient drug distribution systems, poor affordability in low-resource settings, and weak governance structures. However, there were no studies which explored the deeper, contextual narratives and divergent perspectives of key stakeholders across Asia that may underlie or perpetuate these access gaps.

**Added value of this study**

This is the first and the largest multi-country qualitative study to synthesise the perspectives of diverse stakeholders across Asia on barriers to accessing newly approved antibiotics for multidrug-resistant bacterial infections, including extensive viewpoints from the pharmaceutical industry. Participants were selected from countries with varying multidrug-resistant bacterial infection burden–Sri Lanka, Thailand, Cambodia, Malaysia, Indonesia, India, Japan, China and Singapore–to yield regionally representative perspectives.

Unlike previous studies that focused on the scientific pipeline, regulatory frameworks, or single-country case studies, our analysis organised challenges into a cascading framework that moves from misaligned mindset to competing interventions, underdeveloped systems, and ultimately, leadership vacuum. It revealed how differing perceptions of urgency and inconsistent priorities across countries had contributed to fragmented approaches and misalignment in addressing inequitable access to newer antibiotics. In the absence of a shared regional agenda, countries relied on temporary workaround strategies rather than investing in collecting unified epidemiological data, sustained antibiotic stewardship, infection prevention and control, and foundational systems such as regulatory capacity. The study also underscored a lack of regional leadership and shared accountability in driving long-term coordinated action. This framing revealed how siloed efforts by global health actors, regulators, clinicians, and industry failed to converge into coordinated, systemic solutions.

**Implications of all the available evidence**

This study moved beyond conventional policy analysis by exploring behavioural, institutional and systemic factors that limit access to newer antibiotics in Asia. By identifying disconnects in priorities, trust, and responsibility across stakeholder groups, this study provided a more nuanced foundation for designing regionally tailored interventions to improve antibiotic access. Drawing from this analysis, we propose five priority actionable areas to guide targeted regional and national action. First, the lack of consistent and transparent data on resistance patterns hampered evidence-based decision-making and regional priority-setting. Second, inefficiencies and misalignment in regulatory processes delayed timely introduction of novel treatments and create duplication of effort. Third, fragmented procurement systems and limited financing mechanisms contributed to inequitable access and overreliance on ad hoc solutions. Fourth, the absence of dedicated leadership structures limits sustained engagement, cross-sectoral coordination, and accountability across countries and institutions. Finally, insufficient investment in diagnostic capacity, stewardship, and infection control weakened the broader health system’s ability to support appropriate use and long-term effectiveness of new antibiotics. Addressing these interconnected gaps requires a coordinated roadmap that integrates policy, governance, and system-level reforms.

## Introduction

The global rise of bacterial antimicrobial resistant (AMR) infections has triggered urgent calls for improved access to new and effective antibiotics, particularly in nations struggling with high infection burdens. The majority of AMR-attributed deaths are associated with severe diseases, such as bloodstream infections and pneumonia especially in hospital settings.^1^ Resistance to reserve-category antibiotics such as carbapenems has led to absence of reliable standard-of-care treatments and has catalysed the development of newer, targeted antibiotics.^2^

The availability, recommended and prescribed antibiotics for treating multidrug-resistant (MDR) infections vary significantly between high-income countries (HICs) and low- and middle-income countries (LMICs).^3^ Newer antibiotics approved by the United States Food and Drug Administration (FDA) and the European Medicines Agency (EMA) in the last decade, such as ceftazidime-avibactam, cefiderocol, ceftolozane-tazobactam, sulbactam-durlobactam and eravacycline, are now recommended treatments in clinical guidelines for the treatment of carbapenem-resistant infections.^4^ However, in many LMICs, older agents such as polymyxins remain the mainstay of treatment despite their well-documented nephrotoxicity and neurotoxicity.^3^^,^^5^ This discrepancy persists even though LMICs bear a disproportionately higher AMR burden including those resistant to carbapenems, thereby aggravating mortality associated with AMR.^6^

Despite significant global investment in the research and development of antibiotics, often supported by targeted initiatives that incentivise the pharmaceutical industry, there remains a critical gap in ensuring equitable access, particularly in LMICs.^7^^,^^8^ Access to newer antibiotics is a multifaceted and complex issue that involves numerous stakeholders, including drug developers, policymakers, regulatory authorities, healthcare professionals, and patients among others. In Asia, where MDR infections are among the highest globally, this access gap is especially acute. Yet, little is known about how these challenges are navigated by those working across the antibiotic supply and delivery ecosystem. To address this gap, we conducted a qualitative study to gather the perspectives of key stakeholders across Asia and identify the barriers and solutions to accessing newer antibiotics for managing MDR infections.

## Methods

### Recruitment and sampling

We conducted in-depth, semi-structured interviews with diverse stakeholders across Asia, identified through professional networks and associations. Potential participants were contacted via email invitation using purposive and snowball sampling methods, incorporating maximum variation to ensure diversity in expertise and discipline.^9^

Eligible participants included stakeholders involved in antibiotic access ecosystem, such as policymakers (POL), regulators (REG), healthcare professionals (HCP), experts from the pharmaceutical industry (IND), global health actors (GHA), health technology assessors (HTA), and patient advocacy groups (PAG). Global health actors included intergovernmental bodies (e.g., WHO), philanthropic foundations, and non-government organisations that influence antibiotic access and AMR policy through funding, research, technical guidance, or coordination. The study is reported according to the Consolidated Criteria for Reporting Qualitative Research (COREQ) guidelines.^10^

### Data collection

The interviews were conducted by a team of five interviewers trained in qualitative research methods, with backgrounds in public health, pharmacy and clinical infectious diseases. To minimise interviewer biases, training sessions were held and interview guide was piloted before data collection to ensure uniformity in interview techniques. Majority of the interviews were conducted by at least two interviewers and notes were taken after each interview. Weekly debriefs during data collection phase reflected on emerging insights and enhanced reflexivity.

A semi-structured interview guide was developed for each stakeholder group based on literature review (Appendix 1). Interviews were conducted either in person or virtually in English. Each interview lasted approximately an hour, was audio-recorded, transcribed verbatim using Otter transcription software (Otter.ai version 3·58·1 (2024), Mountain view, CA, www.otter.ai) and reviewed for accuracy before analysis.

### Qualitative analysis

The transcripts were analysed following the seven-stage framework analysis,^11^ using a pragmatic, applied and systematic methodological orientation with Lumivero NVivo (version 14). First, transcripts were anonymised using pseudocodes. Second, researchers familiarised themselves with the data by re-reading transcripts. Third, two researchers independently coded each transcript line-by-line, then compared the results to assess inter-rater reliability. Discrepancies were resolved by discussion between two coders for consensus, and if still unresolved, by a third researcher. Fourth, a subset of transcripts was inductively coded to develop a coding framework. Fifth, this framework was refined through team discussions and applied to all transcripts for consistent coding. Sixth, coded data were organised into concept maps using Miro visual workspace^12^ to enable comparisons across participants and stakeholder groups. Finally, thematic patterns and relationships were examined to identify key barriers and potential solutions.

Transcription and coding occurred alongside participant recruitment to determine data saturation. We observed that after about 30 interviews, no new concepts pertinent to our objectives emerged, and subsequent interviews largely confirmed the previously identified themes. Therefore, data collection was concluded after 40 interviews. The final draft was shared with the participants to ensure accurate representation, preserve anonymity in quoted material, clarify results and validate the research findings. This process strengthened the credibility and relevance of the findings.

### Ethics approval

The study was approved by the National University of Singapore (NUS) Institutional Review Board (IRB) (NUS-IRB-2024-129) with the first approval on 18 April 2024. Verbal consent was obtained from each participant and audio recorded after the study details were explained at the beginning of the interview.

### Role of funding source

The funders had no role in study design, data collection, data analysis, data interpretation, or writing of the manuscript.

## Results

**Fig. 1:**
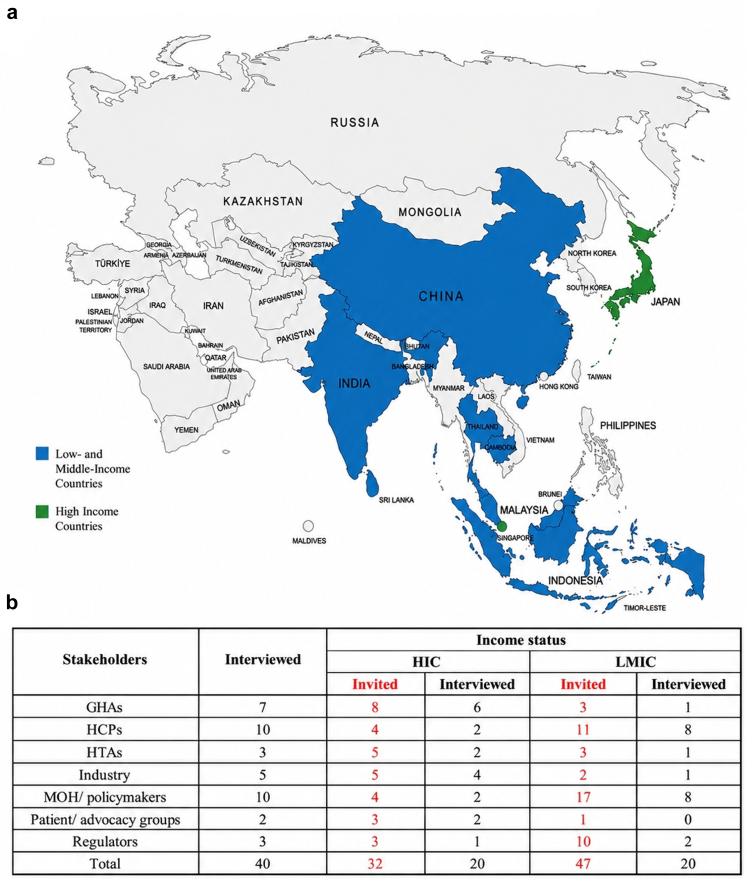
Countries of origin of interview participants. a. Country distribution of participants; b. Stakeholder distribution across countries. HIC: High income country; LMIC: Low- and middle-income country; GHA: Global health actors; HCP: Healthcare professionals; HTA: Health technology assessors; MOH: Ministry of Health.

**Fig. 2:**
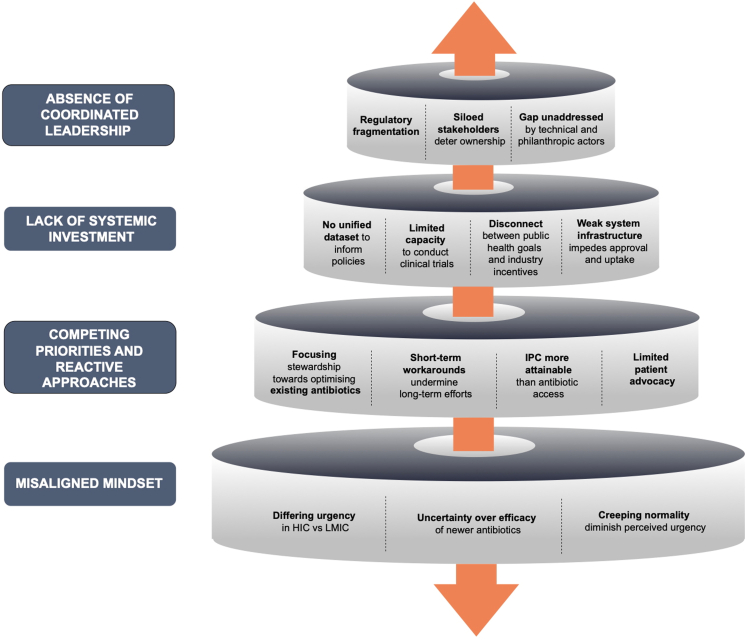
Major themes and subthemes obtained from the interviews.

Interviews were conducted with participants from Cambodia, China, India, Indonesia, Japan, Malaysia, Singapore, Sri Lanka and Thailand (Fig. 1). A total of 79 participants were contacted from April 2024 to April 2025, and 40 interviews were conducted (Supplementary Table S1). More HCPs and POLs were from LMICs (16 vs. 2 from LMICs and HICs, respectively) while more GHAs and INDs were from HICs (2 vs. 10 from LMICs and HICs, respectively). The declined interviews were attributed to reasons such as ‘no response,’ ‘insufficient time,' and ‘the department’s lack of authorisation to provide an interview.’ The latter reason was particularly prevalent among regulators, who were unable to participate without institutional clearance. The analysis identified five major themes and 13 sub-themes (Fig. 2).

### Misaligned mindsets: divergent perceptions of threat and need

#### Perceived urgency of AMR was distant or immediate, depending on context

The perceived urgency of improving access to newer antibiotics for MDR bacterial infections varied between stakeholders from HICs and LMICs, reflecting disparities in how the threat was conceptualised. Participants from LMICs, particularly healthcare providers, expressed the pressing need for newer antibiotics to treat infected patients promptly, citing daily encounters with resistant infections and limited therapeutic options (HTA2(HIC)Q1).

“*If they know the organism is really resistant, they use polymyxins–but not all hospitals [in a LMIC] have access to them. So, basically there's nothing to use*.”–HTA2(HIC)Q1

In contrast, stakeholders not directly involved in patient care or antibiotic development, especially from HICs, perceived MDR bacterial infection burden as a distant threat and questioned the value of investing in access to newer, often more expensive antibiotics. Some healthcare professionals from HICs, remained sceptical of the real burden of MDR bacterial infections, noting that many of these bacteria tended to be non-pathogenic colonisers and not true pathogens. These participants suggested that the AMR burden might be overstated by advocates and questioned whether the call for wider access to new antibiotics was supported by evidence (HCP1(HIC)Q1).

*“I think it [AMR] is a problem. But again, it's a niche problem. And I think, we run the risk of over-dramatising it. So, because most infections actually can be treated, and a lot of times people with antibiotic resistant bacteria isolated are not infected, that they are colonised with these bacteria.”*—HCP1(HIC)Q1

#### Uncertainties over the efficacy of newer antibiotics

Not all participants believed that newer antibiotics were more effective against MDR bacterial infections compared to older options (HCP1(HIC)Q2). These uncertainties were shaped firstly by the design of the clinical trials supporting regulatory approvals. Many of these trials had non-inferiority rather than superiority hypotheses, meaning they showed that the newer antibiotic was no worse than existing options by an arbitrary margin, without proving any advantage. Secondly, these clinical trials were often conducted among patients with less severe MDR infections, such as urinary tract infections. Hence, the generalisability of their results was questioned (HTA1(HIC)Q1).

*“The main thing is that these drugs don't really work … in other words, you're asking me about access to a drug which doesn't work. And a drug which may not work most of the time … I mean, frankly, ceftazidime-avibactam has never been shown superior to Polymyxin or to any of the other comparator agents in the clinical trial. All the trials have been done as the non-inferiority trial.”—*HCP1(HIC)Q2

*“For us, deciding whether to pay for a new antibiotic really comes down to comparative evidence. When we look at newer antibiotics, the data comparing them to what they're meant to replace often just isn't there. So, you can't really conclude that the new drug is better than the old one. And even more than that–how do you justify the price? Sometimes these new antibiotics cost several hundred percent more than older ones. As you rightly mentioned, they're super expensive–way overpriced. So, whether that's a fair price to pay, we would need to evaluate it carefully.”—*HTA1(HIC)Q1

#### “Boiling frog syndrome”—creeping normality diminish perceived urgency

Stakeholders at the country level, grappling with multiple public health challenges, often did not consider AMR among their top priorities. In these participants' view, AMR is a slow-burning crisis that failed to command sustained political or institutional attention. The continued availability of older, albeit suboptimal, treatment options contributed to this perception. As long as some form of treatment existed, even if toxic or less effective, the need for urgent action on antibiotic access remained deprioritised (POL1(HIC)Q1).

“*We are at this slow boiling point, where there isn't that much urgency, but yet, you can see that it is clearly affecting how healthcare is practiced and affecting human outcomes in many patients, right? I have only seen this happen on two occasions for infectious diseases, one of course, for COVID. And the other for HIV in the 1980s and 1990s and drugs became available. For AMR, we have not reached this point. Part of the problem is, and I consider this a problem, is that there are always other options*. *Even if these options are inferior, they are still available. If you have zero drugs or zero options, then yes, it becomes a major problem.”–*POL1(HIC)Q1

#### Limited patient advocacy reinforces the perception of AMR as a low-priority issue

Patient advocacy groups described limited exposure, communication channels, and inclusion in AMR-related dialogues, which constrained their ability to influence antibiotic access agendas. The absence of strong, visible patient voices and resulting lack of public pressure on decision-makers reinforced the perception that AMR was a technical or clinician-only concern. Participants highlighted how outreach efforts often targeted professional audiences, with minimal engagement of the broader patient community, even in settings heavily affected by resistant infections (PAG2(HIC)Q1). This weak public engagement contributed to the broader mindset that AMR is a distant or background threat, rather than an immediate, people-centred crisis warranting policy attention.

*“Not enough, not enough [information about AMR is being shared with patients]. Because it might be at a level where a bunch of doctors are speaking at a seminar. But a total of 100 patients attend while there are 3000 patients affected … so not enough information is being shared. And that's clinicians speaking at that one platform. And that's it.”–*PAG2(HIC)Q1

### Fragmented interventions: competing priorities and reactive approaches

#### Focusing stewardship of existing antibiotics seen as a more immediate priority

Both clinical and industry stakeholders recognised antibiotic stewardship as foundational, not only for preserving existing antibiotics, but also for protecting their long-term efficacy. Healthcare professionals highlighted the consequences of poor antibiotic stewardship in driving resistance patterns. These participants voiced the concern that widespread use of carbapenems to treat third-generation cephalosporin-resistant GNB infections had led to increasing carbapenem resistance. In many countries, a lack of access to FDA-approved, promising newer antibiotics, such as cefiderocol and ceftazidime-avibactam left few antibiotic options (often polymyxins) for treating carbapenem-resistant infections (HCP7(LMIC)Q1).

*“The issue is that agents like cefiderocol, which can cover most of the Gram negatives or ceftazidime-avibactam, which could again work in a similar way, are not yet widely available. The second thing is the significant issue of cost. For example, if you take ceftazidime-avibactam, till Pfizer had the patent, which is about a little more than a year or two, ceftazidime-avibactam was quite expensive for regular use for all patients. So, secondly, there is an issue of cost.”*–HCP7(LMIC)Q1

Similarly, without robust antibiotic stewardship, participants expressed apprehension about lower pricing or broadening distribution as these could, in turn, exacerbate irrational use and resistance development (GHA7Q1).

*“I don't think that the priority in most of this country is about access to new antibiotics. I think the priority is about ensuring that the existing antibiotics are adequately used.”–*GHA7Q1

#### Preventive, non-pharmaceutical solutions viewed as more attainable than expanding antibiotic access

The interviews revealed a divergence in global priorities: while HICs tended to be focused on developing and procuring new antibiotics, LMICs more often prioritised non-pharmaceutical, preventive solutions (POL10(LMIC)Q1). Few stakeholders proposed that infection prevention and control (IPC), alongside Water, Sanitation and Hygiene initiatives, would be more effective in alleviating AMR burden than improving access to newer antibiotics. They emphasised that building IPC capacity and preventing healthcare-associated infections would be more cost-effective and sustainable in LMICs, hence prevention initiatives should be prioritised over accessing curative antibiotics. There was a need to weigh the opportunity costs of investing in premium-priced newer antibiotics against pressing gaps in human resources, diagnostics, and basic infrastructure.

*“I think IPC (infectious prevention and control) is not prioritised at a high level. I think that when we talk about the AMR of high-income countries, they always put a new antibiotic at the top. ‘Where are the new antibiotics? Put more funding into the analytics’. Which is okay, which is good. You should put something about that, but you should not forget IPC and save lives that you can save now … if it's Cambodia, Laos, Africa, I would push sanitation and public water as priority.”–*POL10(LMIC)Q1

#### Short-term antibiotic access workarounds undermine long-term accessibility efforts

The capacity of publicly funded hospitals to procure newer antibiotics remained inadequate, constrained by both affordability and unpredictable trends in the incidence of MDR bacterial infections (HCP6(LMIC)Q1). A few participants mentioned that unregistered newer antibiotics could be obtained on a named patient basis, subject to specific institutional requirements, through alternative pathways. Firstly, the manufacturing company could grant access on a compassionate basis. Secondly, economically privileged patients who could afford the freight costs of antibiotics could obtain them overseas, often paying out-of-pocket as these unregistered antibiotics were not covered by insurance schemes. In these scenarios, the prescribing clinician must accept legal or professional responsibility for any adverse outcomes. The healthcare providers felt that these alternative methods were not only administratively burdensome but also delayed timely treatments for critically ill patients.

*“There were about, I think, 90 patients who requested access for this drug across xx [country]. And finally, the regulatory approvals, the number of patients that use the drug, the number of clinicians that got access finally is approved 38. Because these are very sick patients, quite a few of them died.”*–HCP7(LMIC)Q2

*“They [public hospitals] procure the medication at the beginning of the year. But if you use it [ceftazidime-avibactam] up, then that's it. That's the end of the story. Because you are going to go back to polymyxin B based therapy or Polymyxin based therapy for this patient.”–*HCP6(LMIC)Q1

### Lack of systemic investment: weak infrastructure and evidence base

#### Lack of unified, cross-border dataset capable of meeting diverse stakeholder requirements

All participants indicated that there is an urgent need for a systematic and coherent data collection framework to quantify the burden of MDR infections. While national surveillance systems existed in some countries, these were often fragmented across institutions, inconsistent in methodology, or lacked interoperability between regions (HTA3(LMIC)Q1). Most surveillance data relied solely on microbiological results without linkage to clinical outcomes, making it difficult to assess the severity or impact of infections caused by resistant pathogens. Global estimates, such as those from the Global Burden of Disease study, were viewed as useful for advocacy, but several participants felt these models were based on limited real-world data from Asia, and therefore lacked credibility for local decision-making (HTA1(HIC)Q1). Many participants highlighted issues such as inadequate laboratory infrastructure, inconsistent sample collection, and poor data management, which lead to fragmented data and an unclear understanding of the clinical and economic burden of MDR infections. This weakened the case for prioritising newer antibiotics in national health strategies (HCP8(LMIC)Q1).

*“When you do a case study, you need to compare with a reference, it’s like a checklist as well. In HTA, we don’t have that guideline or reference case for antibiotic evaluation. And the lack of this reference case of guidelines is not because people do not see the importance but because it is really difficult. Many teams have tried to address this by developing recommendations, guidelines, and references, but all have failed so far*.”- HTA3(LMIC)Q1

*“… but it's also how much robustness or weight we can give to such data. Yeah, so I think it[evidence] has to be case by case, assessed by what kind of data that is being received*.*”—*HTA1(HIC)Q1

*“I think that cost is the problem [to getting essential antibiotics]. But the real problem is the lack of research in this field. So if we have good evidence to prove to the policymaker that it will work, we can order this [cefiderocol and ceftazidime-avibactam] drug. Every year we had to make a budget plan to the government and the budget plan based on the need and based on the number of the different types of the drugs that we use. We don't have much research evidence to show that we really need this drug in the community or in the hospital.”–*HCP8(LMIC)Q1

Similarly for pharmaceutical companies, companies were unable to forecast demand or justify market entry without robust data (IND3Q1).

*“If the demand is not sufficient, then the investor will face difficulties in the future. So it's really difficult to invest huge money in huge facilities. If they really want to invest money in these facilities, they need to understand how much or how much products the world will need, and it should be constant, but it's very difficult to predict”–*IND3Q1

In addition to technical limitations, participants also highlighted political and financial barriers to improving AMR surveillance. Several noted that while many countries had formalised AMR National Action Plans (NAPs), these strategies often remained underfunded and unimplemented. Despite allocations on paper, governments had not disbursed necessary funds for operationalising data systems or strengthening laboratory capacity (POL9(LMIC)Q1). This led to a vicious cycle: limited investment yielded poor quality or incomplete data, which in turn made it difficult to justify further funding or action. The result was systemic inertia that perpetuated the status quo.

“*So far, we use only the international partner budget and have not used the national budget yet. But this year and next year, I proposed an allocation of the national budget to support the AMR [national action plans] … We do not know [the outcome] yet. On the way to the negotiation.”* POL9(LMIC)Q1

### Limited capacity to conduct clinical trials across Asia

Participants recognised that robust and locally relevant clinical evidence was essential for driving antibiotic access decisions. However, they reported inadequate clinical trial capabilities especially in many LMICs. The suboptimal health research infrastructure hindered pharmaceutical companies from conducting their trials due to prolonged trial timelines and increased costs, thereby discouraging registration and market entry in these countries. Participants noted that small pharmaceutical enterprises, responsible for about 80% of new antibiotic development, often lacked the funding and resources to conduct trials and meet individual countries’ regulatory requirements and registration (IND3Q2, GHA6Q1)

*“I think the biggest challenge we have for small companies is getting a meaningful clinical trial done at a reasonable cost. If I run a trial through a CRO (contract research organisation) at … let's say 200 patients in an ICU. I'm sure this was a $50 million trial, right, with all the CRO costs and everything behind it, and now you want to have that for 8 to 10, different drugs, you're looking at a half a billion and these are abbreviated clinical development pathways.”–*IND3Q2

“*It would be great to have some kind of alternative pathways for doing clinical trials in a more cost efficient manner. And I think there’s a benefit in being able to do them in certain countries where burden is quite high and patients are easier to enroll from that perspective*”- GHA6Q1

As a result, health technology assessment bodies, regulatory agencies and healthcare practitioners felt industry-sponsored trial data were limited in both geographical representation and infection syndrome scopes, as they did not include comparisons against local standard-of-care therapies (HTA1(HIC)Q2). For example, many newer antibiotics were approved for use in uncomplicated infections such as urinary tract infections, which offer limited insight into drug efficacy for more complex and resistant infections commonly seen in LMICs. The health technology assessors also highlighted the limitations in conducting cost-effective evaluation of antibiotics due to their inherent complexities.

*“We generally look at published trials from the companies, because any local data, like real world evidence, is more supplementary nature. We focus on whatever published evidence is out there, because they are typically sponsored by the companies manufacturing and that's the best available evidence we have. Some of these local data on antibiotic resistance patterns and all, we will take into consideration when we also look at establishing the clinical need, the place in therapy for the treatment, but they are not used to establish the comparative evidence.*”–HTA1(HIC)Q2

### Weak health system infrastructure impedes approval and assimilation of new antibiotics in local markets

Participants from regulatory agencies in LMICs relied on small teams to evaluate clinical trial data across multiple drug categories, resulting in backlogs and delays (REG1(LMIC)Q1). They highlighted the importance of communication and aligning priorities with ministries of health for faster review of high-priority drugs. However, they also emphasised the need to preserve their independence against external pressures that could compromise rigorous reviews.

*“Unfortunately, the approval of medicines, we have a huge backlog. From 2019 to 2024, those four years, there's a backlog. We have a reason for the backlog, so one is the shortage of staff and the poor expertise of the regulatory staff. So, because of the running out of staff, I'm doing the registration of the medical devices, of the medicines, and of cosmetics.” -*REG1(LMIC)Q1

On the other hand, industry representatives voiced concerns about intellectual property protection and navigating country-specific regulatory requirements, ranging from demonstrating pharmacokinetics and pharmacodynamics in local populations to complying with distinct manufacturing and distribution standards (IND2Q1).

*“So how the relationship between the countries is one of the points for us, that is true. And second one is about the IP (intellectual property) in the country. But if we try to enter a developing country, some countries are not good at protecting IP. In this case, we cannot enter. We cannot.”–*IND2Q1

Even after regulatory approval, integrating newer antibiotics into clinical practice was often delayed by systemic limitations. The rollout of such antibiotics was logistically complex and resource-intensive, requiring staff training, supply chain readiness, and stewardship protocols. Newer antibiotics such as ceftazidime–avibactam required specialised diagnostics to confirm resistance mechanisms and devices for prolonged infusions, posing logistical challenges (HCP2(HIC)Q1). Participants, even from HICs, highlighted the lack of appropriate diagnostic tools and limited capacity, resulting in lengthy preparation timelines for safely integrating these antibiotics into practice (HCP7(LMIC)Q3).

*“But then, until the official either FDA (Food and Drugs Administration) or CLSI (Clinial and Laboratoratory Standards Institute) or EUCAST (European Committee on Antimicrobial Susceptibility Testing) breakpoints and a standardised methodology, it's hard to steward or decide who is appropriate for this antibiotic. For example, ceftazidime-avibactam with aztreonam, there's no breakpoint yet, unless you infer from aztreonam breakpoints. And there's also no standard method currently, so there's no device. There's no CLSI or recognised method or breakpoint.”–*HCP2(HIC)Q1

*“There are no good ways to sort of suspect who has carbapenem resistant infection. And if the diagnostic setup is not very good, your diagnosis and identifying these patients itself is a problem. So that brings in a huge amount of challenge for a public hospital to sort of identify these patients.”–*HCP7(LMIC)Q3

Healthcare professionals faced ethical dilemmas when deciding who should receive newer and costly antibiotics as there were no local guidelines or antimicrobial stewardship programmes (HCP9(LMIC)Q1). Without regional or LMIC-focused recommendations, most physicians found it challenging to make prescribing decisions based on evidence from HICs, where newer antibiotics were unanimously preferred (HCP7(LMIC)Q4).

*“You have a very limited stock, sometimes you also don't know which patient you should give [the medicine to], you see? In the end, you don't use it because you feel like you do not know-which patient should I start? In the end, you will go back to polymyxin and colistin. Same goes to ceftazidime-avibactam. Six months already in this hospital, but none of the doctors want to start because we have limited stock and they are also confused which patient to give.”–*HCP9(LMIC)Q1

*“If you look at all global literature, from IDSA (Infectious Diseases Society of America) or ECCMID (European Congress of Clinical Microbiology and Infectious Diseases) they would say they would clump Klebsiella and E. coli together. And then say that for* Enterobacteriaceae*, say, you use ceftazidime-avibactam with confidence, this will be one of the first line drugs to use. But in [a LMIC country] you can't translate that because E. coli will not work most of the time but for Klebsiella it will work phenomenally well. So in [a LMIC country], when you come up with an empirical or even a definitive therapy, waiting for molecular confirmation, you have to use caz-avi (ceftazidime-avibactam) plus aztreonam. Then with Pseudomonas, your first claim is again, ceftazidime-avibactam, ceftolozane-tazobactam, and things like that, which won't work here, because of the MBL (metalo-betalactamase resistance) rates in Pseudomonas. So we need a lot of caution in translating these guidelines across.”–*HCP7(LMIC)Q4

Health technology assessors mentioned that very little of their evaluation budgets (often <10%) were allocated for new antibiotics, which limited cost-effectiveness assessments and slowed their addition to national formularies (HTA1(HIC)Q3). In the absence of national subsidies, integrating these drugs into clinical practice became even more challenging.

*“We have certain criteria, whether it's high clinical need evidence and then prioritise certain topics. There has never been like a big impetus for us to sort of look at antibiotics all this time, in part because a lot of antibiotics that are used in the inpatient setting, there's [insurance] coverage for inpatients. … As we shared, there's only one team looking at all the topics, then it's about prioritising which topics, because there's only three meetings per year and only a fixed number of topics each year.”-* HTA1(HIC)Q3

#### Disconnect between public health goals and industry incentives reflect systemic underinvestment in antibiotic access

Industry stakeholders described a fundamentally misaligned and unsustainable system for antibiotic development and access. Unlike other therapeutic areas, the economic model for antibiotics is shaped by high research and development costs, unpredictable demand, complex regulatory pathways, and expensive market entry requirements (GHA6Q2). New antibiotics, often produced by small or mid-sized pharmaceutical enterprises, must be used sparingly to preserve their efficacy, making volume-based pricing models unworkable. This structural disconnect between public health goals and commercial incentives was seen as a core system failure that continues to undermine access and affordability.

*“Schemes that improve predictability are really important. Pooled procurement agencies e.g., Global Fund and GDF work because supply and access is standardised, despite there still being a need for local registration … I think the other problem is just the uncertainty of the market, which is, maybe there’s not great demand forecasting. It’s kind of the equivalent in the antibiotic space and so a company doesn’t know what the demand will be this year, next year, or 10 years from now. It’s going to look very different and not just because the science changes, but because a country or a hospital itself doesn’t necessarily know*”–GHA6Q2

Participants noted that low or unreciprocated demand signals have discouraged sustained investment in antibiotic pipelines, raising concerns about the long-term viability of antibiotic discovery and marketing programmes (IND5Q1). Several companies faced substantial difficulties in maintaining or rebuilding research teams after exiting the antibiotic field, underscoring the fragility of capacity in the absence of systemic support.

*“So the biggest casualty of our poor antibiotic sustainability is going to be the research teams, discovery teams, and once they are gone, your source of the new antibiotic itself is gone. There will be no discussion of access.”-* IND5Q1

In an attempt to recoup costs and reduce risk, industry stakeholders noted that the majority of commercialisation activities for newer antibiotics flowed into HICs despite LMICs needing them more. Pharmaceutical companies naturally gravitated towards HICs where established regulatory frameworks, reliable clinical trial infrastructures, and greater purchasing power facilitated more predictable return on investment despite lower AMR burden (IND4Q1).

“*The driving force for companies or industries is to go first to the FDA (Food and Drug Administration), because the access to this is more straightforward, than in Europe. And we’ve seen that recently, some new antibiotics that have been approved have never been licensed in Europe. And that’s simply due to the cost of going to each one of these countries separately … 75% in the market, I would need three approvals, basically, whereas then to gather the other 25% you would have to go through each country separately. That’s the driving force we need to get as a company. We need to show the investors, to everyone, that we’re going to the current markets. But this doesn’t necessarily mean that this is where the medical or unmet medical need really is. And that is a bit of the conundrum that we’re living in–that we need to go to the market, yet the medical need is often elsewhere*”–IND4Q1

### Absence of coordinated leadership: No one driving antibiotic access agenda

#### Siloed regional and sectoral approaches to antibiotic access limit coordinated ownership

All participants agreed that challenges of antibiotic access could only be addressed through a collaborative, multi-stakeholder approach. However, there were no channels for establishing these dialogues or work plans currently. There was a void of ownership by an entity that could lead coordination efforts at the national and regional levels (POL2(HIC)Q1). Many participants mentioned that this ownership needed to come from the ministries of health (GHA1Q1).

*“There is not a central authority that dictates the AMR policy response. It's usually a conglomerate of different players and actors that try and work together. Even in the US, even the UK, you have more emphasis on this. It belongs to a lot of stakeholders, and I don't know whether there's one person who is full, like, accountable for these problems.”–*POL2(HIC)Q1

“*I think, ultimately, it [access] has to be a priority of the Ministry of Health. We help develop public or private health programs which ultimately will sustain after we [GHA] intervene, they tend to be owned or registered by the government.*”—GHA1Q1

#### Regulatory fragmentation across countries

Many stakeholders supported harmonisation of drug approval processes to share resources and reduce duplication. However, the logistics of harmonising regulatory processes were constrained by linguistic barriers and a lack of formal frameworks for collective action (GHA3Q1). The success of such initiatives required much investment in building trust and reliance across agencies (GHA6Q3).

*“I think when we spoke to a lot of the regulatory agencies, they feel typical things like understaffed, underfunded, they may start to band together, but I think there needs to be like champions within to drive that from an AMR focus because they're not going to have a specific AMR regulatory person- they do general approvals”.–*GHA3Q1

“*Harmonisation is helpful, I think, from the point of each new market launch being smaller incremental works rather than having to start from scratch. From the perspective of everyone, there has to be a common understanding of what’s required to prove that this product is effective, it’s safe, it’s quality assured, etc. That there aren’t different ways of assessing this*”–GHA6Q3

#### Governance and coordination gaps remain unaddressed by global health technical and philanthropic actors

GHAs supported the various stages of antibiotic development pathway but, according to industry participants, that support was not enough to ensure antibiotic access (IND4Q2). Initiatives such as Combating Antibiotic-Resistant Bacteria Biopharmaceutical Accelerator (CARB-X), Global Antibiotic Research and Development Partnership (GARDP) and AMR Action Fund addressed research and development in innovative and novel fields or late-stage development gaps, but they provided comparatively little support for marketing, regulatory filings, and procurement efforts in LMICs. Small and medium-sized pharmaceutical companies found commercialising a new antibiotic in high-burden countries a critical and challenging step, and voiced the need for guidance or a coordinating agency.

“*We should keep it in mind that those (encouragement to novel conceptual interventions such as vaccines, phages, diagnostics by CARB-X) will not yield you a viable product which can be used in the patient, where you can save the life of today's patients or tomorrow's patients … So the question is, are our funding agencies, supporting agencies maintaining a balance between supporting a product which will come 10–15 years down the line, or whether they are supporting a project which can become a lifesaving project in just the coming three or four years.”–*IND5Q2

“*And so, we are dependent on xx (GHA), because their goal would be to help bring companies forward and bring new drugs to the market, Right? That should be their goal. So we are all somewhat looking for support from them. But keep in mind, that the support is generally very small. They haven’t invested very much in any company, and so it’s not a lifesaver*.”–IND4Q2

## Discussion

Our study identified major ambiguities and conflicts around perceived needs, potential solutions and investment priorities for improving access to newer antibiotics in Asia. There was a cascading set of barriers from ground up, beginning with misaligned mindsets around AMR and extending through fragmented interventions, systemic underinvestment, and ultimately a lack of coordinated leadership. Participants described how divergent perceptions of AMR urgency, shaped by clinical visibility, data availability, and stakeholder roles, influenced which interventions were prioritised. These competing priorities led to siloed efforts, short-term fixes, and reactive strategies, rather than cohesive, long-term planning. At the system level, persistent gaps in infrastructure, data, clinical trial capacity, and regulatory readiness further undermined the integration of newer antibiotics into care. Despite the presence of influential GHAs, the absence of clear institutional ownership and sustained leadership was seen as a critical bottleneck, leaving the antibiotic access agenda diffuse and deprioritised.

Beyond structural and technical barriers, we were able to uncover deeper mistrust, lack of accountability, and competing narratives that underpin inaction. By making these dynamics visible, our findings explained why well-intended solutions failed to gain traction or produce impact in high AMR burden countries. With no single actor seen as responsible for driving systemic change, efforts remained siloed across stakeholders: clinicians, regulators, procurement bodies, and international NGOs, contributing to limited coordination and reinforcing what was widely perceived as a broader market failure. Unlike vertical programmes in other global health domains, antibiotic access lacks both a unifying narrative and a central institutional driver, leaving it vulnerable to fragmentation and inertia.

This study laid the groundwork for more tailored, politically and institutionally informed strategies to improve equitable access. Firstly, strengthening AMR surveillance capacity and data-sharing platforms, such as A Clinically-Oriented Antimicrobial Resistance Surveillance Network (ACORN) and AutoMated tool for Antimicrobial resistance Surveillance System (AMASS), could help overcome data fragmentation and inform regulatory approval and national subsidy priorities.^13^^,^^14^ Secondly, regulatory harmonisation and pooled procurement through recently launched mechanisms like the ARO alliance for Southeast Asia (ARISE) and WHO’s SECURE initiative can reduce the time, cost, and commercial uncertainty associated with market entry, making it more feasible for manufacturers to register and supply antibiotics in underserved settings.^15^, ^16^, ^17^ Thirdly, advocacy through engaging with the community, professional societies and policy makers are essential to increase awareness and political visibility of AMR.^18^ Fourthly, more efficient clinical trial infrastructure is critical for understanding the local context. Strengthening trial networks by initiatives such as ADVANCE-ID,^19^ and WHO Global Clinical Trial Forum^20^ could support faster approvals of new antibiotics and enable inclusion of priority populations in access plans. Lastly, enhancing diagnostic capacity, promoting antibiotic stewardship, and implementing infection prevention and control measures will prevent infections and reduce reliance on newer antibiotics. Partnerships between governments, pharmaceutical industry and neutral actors such as GHAs, may be essential to catalyse these processes while building long-term institutional capacity.

We acknowledge several limitations in our study. While we sought to capture a diverse range of perspectives across stakeholder groups and geographies, our sample may not fully reflect the heterogeneity of experiences and priorities both between and within countries and sectors. This reflected the scarcity of stakeholders especially for regulators and patient advocacy. To address the lack of participation by regulators, we incorporated their viewpoints from a global regulatory meeting held in Singapore in June 2024 by analysing the meeting's discussions systematically with the same framework used for the interview transcripts. We adhered to a participant validation process, in which participants reviewed findings, to minimise misinterpretation of stakeholder views.

Our findings suggest that improving access to newer antibiotics will require not only technical solutions, but also greater political commitment and mechanisms for cross-sectoral coordination. Addressing these issues will demand more inclusive, context-sensitive approaches grounded in the realities of LMIC health systems and led by actors capable of bridging geopolitical divides and aligning individual incentives with broader public health goals. Future steps should involve creating a detailed roadmap with targeted solutions to expand access to critical newer antibiotics for the populations that need them most.

## Contributors

Shweta R Singh: conceptualisation, data curation, formal analysis, interview conduction, methodology, project administration, writing-original draft; Alejandro Blando-Arévalo: conceptualisation, data curation, formal analysis, interview conduction, methodology, writing-original draft; Atharvi Gupta: conceptualisation, data curation, formal analysis, interview conduction, methodology, writing-original draft; Alec Ann Alissa F. Aligui and Jia Yi Chee: conceptualisation, data curation, formal analysis, investigation, methodology, writing-original draft; Stanton Winter Hor: conceptualisation, data curation, formal analysis, interview conduction, methodology, writing (reviewing and editing); Yiyun Shou, Rathi Saravanan, James Leong, Esmita Charani–conceptualisation, formal analysis, writing (reviewing and editing); Yin Mo: conceptualisation, project supervision, formal analysis, writing (reviewing and editing). All authors had full access to all the data; they have verified the data in the study and accept responsibility for submitting the manuscript for publication.

## Data sharing statement

Anonymised quotes for each major and minor theme and COREQ statement are available in Supplementary Tables S1 and S2.

## Declaration of interests

We declare no conflicts of interest.

## Appendix A Supplementary data

Appendix 1 and Supplementary Tables S1 and S2

Case boxes_ABxAccess
