# Supplementary material for: Barriers to accessing newer antibiotics in countries with high burden of bacterial antimicrobial resistant infections—a qualitative study
Source: Lancet Reg Health West Pac. 2026 Jul 16;72:101917. doi: 10.1016/j.lanwpc.2026.101917 (PMC13400380; doi:10.1016/j.lanwpc.2026.101917)
Supplement: Appendix 1 and Supplementary Tables S1 and S2 [file mmc1.pdf]

## Supplement appendix

Supplement to: Barriers to accessing novel antibiotics in countries with high burden of bacterial antimicrobial resistant infections – a qualitative study

Shweta R Singh, Alejandro Blanco-Arévalo, Atharvi Gupta, et al.

**Contents**

**1. Interview Guide**

1a. Interview Guide for Policy Makers ………………………………………………………. 3
1b. Interview Guide for Regulators ………………………………………………………... 4
1c. Interview Guide for Healthcare Professionals …………………………………………… .6
1d. Interview guide for Industry Experts ……………………………………….…………. .8
1e. Interview Guide for Non-government Organisations ……………………………………. 10
1f. Interview Guide for Health Technology Assessors ………………………….………….12
1g. Interview Guide for Patient and Patient Advocacy Groups …………………………...…..13
**2. Results**
Table S1. Themes, sub-themes with representative quotes ……………………..…………...15
Table S2. COREQ statement ………………………………………………………………..18

## 1. Interview Guide

### 1a. Interview Guide for Policy Makers

**Baseline questions:**

1. How many years have you been working in this field and organization?

**AMR burden of disease:**

1. What is the greatest bacterial burden of disease in your country? (e.g. Lower respiratory tract infections, bloodstream infections)
2. What is the antimicrobial resistance breakdown of these diseases?
3. Is the ministry in your country prioritizing the search for alternative treatment for difficult to treat gram-negative bacterial infections?
4. How could cefiderocol and/or ceftazidime-avibactam alleviate this disease burden?

**AMR key stakeholder and roles:**

1. In your perspective, what role do Ministry of Health (MOH) or policymakers play in gaining access to new generation antibiotics in your country?
2. What are some national AMR coordinating groups or groups with similar interests in your country?
    a. What do they do or what is their role, who do they consist of?
    b. How often are the meetings scheduled?

**Antibiotic access:**

1. How are the groups mentioned above addressing access to cefiderocol and/or ceftazidime-avibactam? What are the challenges/barriers?
2. Can you provide an overview of the key stages or processes involved in the access to novel antibiotics (specifically to cefiderocol and/or ceftazidime-avibactam)?
    a. Which of these steps you find to be the most challenging. If applicable, how have you or your organization overcome these challenges?
    b. Examples
        i. Must review finance,
        ii. Budget,
        iii. Relationships with pharmaceutical companies,
        iv. Work with regulatory agencies, and
        v. Consider treatment guidelines.
3. What strategies are currently in place to increase / provide access to cefiderocol and ceftazidime-avibactam in your country? For example,
    a. Incentivizing pharmaceutical companies,
    b. Affordability for patients, and
    c. Antibiotic stewardship programs.

**Additional questions:**

1. In your perspective, what roles does international collaboration play in addressing the global challenge of antibiotic resistance and ensuring equitable access to novel antibiotics?
2. From a supply chain standpoint, how are logistical challenges (if any) in ensuring the efficient distribution and availability of cefiderocol and ceftazidime-avibactam managed?

**1b. Interview Guide for Regulators**

**Baseline questions:**

1. How many years have you been working in this field and organization?
2. What is your involvement in antibiotic approval or marketing authorization application (MAA) and distribution of antibiotics?

**Approval Process Overview:**

1. Can you provide an overview of the regulatory approval pathway for new antibiotics, from initial submission to market authorization? e.g. different steps
    a. Do you refer to other country's regulatory agency review for reference?
2. How long does it typically take from the initial submission to the response and then to the approval of an antibiotic?
    a. Is this timeline the same for other drug classes?
3. Are there any accelerated approval pathways specific to antibiotics?
    a. Is there any priority approval process for antibiotics vs other drugs?
4. How many people are involved in the approval of a drug?
5. Is there any person-to-person contact with pharmaceutical companies during the approval process?
6. What are the main reasons for rejecting the approval of an antibiotic?
    a. What happens if the rejected drug has been approved by other countries?
7. If a submission is rejected, can the company appeal?

**Evaluation Criteria and Special Considerations:**

1. What role do clinical trials play in the antibiotic approval process?
    a. What are the criteria in these trials?
2. What are the challenges specific to evaluating the safety and efficacy of antibiotics compared to other drug classes?
    a. e.g. some of the data/trials have insufficient data or concerning data
    b. Are there unique considerations in choosing an indication for the approved drug, such as age-specific concerns or dosage?
3. Does your agency have the relevant regulatory technical capacity to review novel antibiotics?
    a. e.g. insufficient manpower, or insufficient subject matter expertise
    b. If not, does your agency engage external regulatory experts independent of the pharmaceutical companies?

**Challenges and Improvements:**

1. Do you see any major limitations in the current regulatory framework for antibiotics that can be improved to accelerate the process?
2. In your opinion, what else can be done to make the process fair and free of any influence?
3. Do you review all the antibiotics that enter the international market/existing registration in other countries, or is it reviewed only when an individual/company requests the review for your individual country?
4. Do you also assess or consider the potential impact of the antibiotic on antimicrobial resistance (AMR) patterns before approval?
5. Are there any incentives aimed at promoting the development of novel antibiotics?
6. Is there any opportunity for regulatory reliance and cooperation involved in the marketing authorization application (MAA) of new antibiotics in your agency

**Post approval:**

1. Do you review the distribution plan of the drug in the country? What is the time frame from approval to country wide distribution?
2. How do you establish the recommended price of the new drug? Is it a negotiation between the pharma industry and the agency? Is there any agreement in lowering the price based on the potential use of the drug? e.g. drug volumes, subsidies

**Monitoring:**

1. How do you ensure the post-approval surveillance and monitoring of antibiotics?
2. Is it mandatory to provide an antimicrobial stewardship plan in the MAA to prevent antibiotic abuse?
3. What is the course of action if the approved antibiotic has some unexpected and significant side effects?

**Regulatory:**

1. What does your department do and what work have you done on antibiotics in terms of regulation, quality etc
    a. Who else or other departments do you work with typically, and for what work?
2. How is your relationship with national regulatory authorities,
3. What is your view on the current regulatory processes in your countries. Is it efficient, could it be improved?

4. What do you think of the concept of a regional regulatory approval? Is World Health Organizations (WHO) working on something along these lines?
5. How often do you meet with pharmaceutical companies to discuss regulatory barriers and what do these discussions involve?

**Access**

1. What is your view with regards to antibiotic access and how does WHO enable access to countries?
2. Do you see countries facing challenges of receiving drug/antibiotic applications from industry?
    a. What are some solutions or work that WHO is doing to address this?

**Quality**

1. Do you see a lot of counterfeit antibiotics (e.g. ceftazidime avibactam) and over the counter antibiotics use?

**General**

1. Do you work at all with Health Technology Accessors (HTA) of your countries?
    a. If so, how? Do they play a significant role in antibiotic access?
2. Do you have anything else to share regarding this interview and antibiotic access?

**1c. Interview Guide for Healthcare Professionals**
**Baseline questions:**

1. How many years have you been a physician and what is your current affiliation?
2. What various AMR/ID related committees or groups do you participate in?
    a. What are these groups' roles?

**Questions:**

1. What are your thoughts of the current situation of antimicrobial resistance against gram-negative bacterial infections in your country?
2. On average, how many patients with this resistance (carbapenem-resistant *enterobacteriaceae, pseudomonas, acinetobacter*) do you encounter per month?
3. Do you have any concerns about using the currently available antibiotics (e.g. polymyxin & side effects) for difficult to treat gram-negative bacterial infections?
    a. What antibiotics are you currently prescribing for the above? (Please list down for Carbapenem-resistant *Enterobacteriaceae*, *Pseudomonas Aeruginosa, Acinetobacter Baumanii*
    b. Do you wish/ hope you have better alternatives, why?
4. When did you become aware of more recent last-line antibiotics like cefiderocol and ceftazidime-avibactam? (e.g. already knew at clinical trial stage, vs knew once it was FDA approved and used in US)
5. What are your opinions regarding these antibiotics?
    a. e.g. clinical evidence, non-inferiority, access (or lack of)
    b. Would you prescribe them today or wait for more data?
6. Are these antibiotics currently available in your country?
    a. If so, when did they become available?
7. Do you perceive a demand for cefiderocol and ceftazidime-avibactam in your country?
    a. For example, out of every 10 patients (who have an antibiotic-resistant infection), how many would likely require these new antibiotics?
    b. For example, how many difficult to treat gram-negative bacterial infections would you get in your hospital per month/year?
    c. What are the resistance rates for Carbapenems in your hospital? Are they on a worrying rising trend?
8. Do you recall any significant events related to these antibiotics that you have witnessed in your country? (e.g. clinical trials, regulation, donations)
9. Have you faced any challenges in gaining access to these antibiotics? e.g.

    a. Hospital-level
        i. What additional steps are required to prescribe these antibiotics? (e.g. therapeutics board / infectious disease approval)
        ii. Are there any issues with medical board / hospital formulary approval/incorporation?
        iii. Are there any issues on delays due to microbiology e.g. culture sampling, laboratory processing and results?
        iv. How long does it typically take from the time a prescription is requested until the antibiotic reaches the patient?
        v. What are the implications of delays in patients receiving the antibiotic?
        vi. What specific steps, if removed or streamlined, could significantly expedite access to these antibiotics?
        vii. Do patients sometimes refuse these new antibiotics due to their higher price?
        viii. Any supply or exemption access issues?

    b. Government-level
        i. Has the government shown interest in providing access to these antibiotics?
        ii. Has the government shown interest in subsidizing these antibiotics?
        iii. What measures could be taken to encourage government subsidy or increased availability of these antibiotics?
10. Is there an antibiotic stewardship program in your hospital?
    a. What changes have you observed before and after the implementation of the stewardship program?
    b. Is your hospital in a good situation to properly diagnose drug resistant gram-negative infections, prescribe and monitor Cefiderocol and Ceftazidime-Avibactam?
11. What challenges do you anticipate if cefiderocol and ceftazidime-avibactam are more readily available?

12. Who do you believe should be engaged in the discussion for enhancing the availability and accessibility of these antibiotics? For example, other stakeholders, organisations?
13. Before we conclude, is there anything else you would like to share that you think might be relevant to our discussion?

**1d. Interview guide for Industry Experts**

**Baseline questions:**

1. How many years have you been working in this field and organization?
2. What is your involvement in antibiotic approval or marketing authorization application (MAA) and distribution of antibiotics?

**Analyzing the market:**

1. What are the factors considered in choosing a region/country for market entry?
    a. What are the common challenges, for example government policies or regulatory authorities?
2. What are the factors considered in choosing a region/country for distribution?
    a. What are the common challenges, for example affordability, burden of disease, regulatory hurdles?
3. How do economic or other factors in LMICs influence the company's strategy for pricing?
    a. What factors played a role in their consideration?

**Interaction with regulators:**

1. What are the general steps in getting a drug approved in a new country?
2. What regulatory challenges did the company face when introducing antibiotics in LMICs?
3. How do you try to open the drug to more indications than the ones assessed in the pivotal clinical trials?
    a. If they don't seek more indications after clinical trials, why?
5. How do you negotiate the pricing with different regulatory agencies?
6. Do LMICs have special conditions for pricing?

**Supply chain, distribution and manufacturing:**

1. In the context of LMICs in South-East Asia, how does the company ensure proper supply and storage of antibiotics? (e.g. cold chain, cold rooms, storage conditions)
    a. What factors are considered for intra-country distribution of the antibiotic?
2. What are the existing infrastructure constraints for adequate manufacturing, supply chain and distribution network?
    a. Do you try to use partner companies to assist with manufacturing, or distribution, why?
    b. What are your thoughts with regards to allowing local manufacturing of your product, example for Southeast Asia?

**Interplay with other stakeholders:**

1. What initiatives does the company undertake to educate HCPs and the public about the use of these antibiotics in developing countries?
2. What percentage of the company's annual budget is expected in promotion and education about the drug in LMICs?
3. How did you decide to have exclusive rights of Cefiderocol to Singapore, Vietnam and Indonesia? What are the factors in decision making?
4. Do you have any stewardship plan / requirements for your antibiotic? Please elaborate.

**Gathering Feedback:**

1. How does the company gather feedback about the limitations in use and other challenges in prescription from HCP in LMICs?
2. How does the company process and incorporate this information into strategies and action?

**Long term plans/ strategies:**

1. Would you like to add anything that the company would like to do differently in future? (regarding their current antibiotic or for future development of antibiotics)
2. Would you like to add anything else relevant that we have missed?

**1e. Interview Guide for Non-government Organisations**
**Baseline questions:**
1. How many years have you been working in this field and organization?

**Role:**
1. What is your organization's vision / mission for antibiotics? For example, access, R&D, new antibiotic development?
2. Could you elaborate on the specific role your organization plays in enabling access to antibiotics in the countries of our interest mentioned earlier? If not working in these four countries, any other country's example?
3. What are your organisation's countries or regions of focus? Why did you decide on these countries?

**Country Market Analysis:**
1. What considerations does your organization include while evaluating antibiotic accessibility in the countries of our interest (or any country)? Please illustrate with any country cases or examples in terms of
    a. Market demand: market size, consumer needs, epidemiological factors
    b. Supply chain dynamics: distribution networks, local manufacturing and import practices.
    c. Regulatory landscape: compliance, quality assurance
    d. Pricing and affordability: pricing models, healthcare financing structures, government subsidies
    e. Competition analysis: presence of local and international manufacturers, market entry barriers
    f. Patient behavior & practices
    g. Competence in technology
    h. Stakeholder collaborations
    i. Data accessibility and surveillance
    j. Antibiotic StewardshipProgram
    k. Public health need, country readiness
    l. Previous work in country
4. What is your assessment or conclusion of antibiotic availability and access in the countries of our interest? For example, hard market to penetrate, low demand, easy to work with MOH / local regulatory authority. Are theer any country cases or examples?
5. What are the primary challenges contributing to poor access to last line antibiotics in these four countries? Please rank the top five challenges in order of highest priority.
6. Do you have any suggestions or solutions to address the challenges in the previous question?

**Enabling Access:**

7. *(ask if they have not provided examples yet)* Have you previously undertaken initiatives or helped improve drug and/ or antibiotic access in these four countries, and if so, could you provide examples or specific projects? If not in these four countries, what about other country examples?
    Clarifications:
    a. Project details and overview
    b. Timeframe
    c. Rationale
    d. Demand
    e. Were the initiatives conducted independently? Specify involvement (if any). E.g. local healthcare organizations, government agencies, or other stakeholders
    f. Outcomes achieved from the specific project mentioned (does it align with your company's goals, any success stories, have the lessons learnt influenced the evolution of your organization's approach?)
    1. Is your organization involved in any initiatives/efforts in providing the public information/education about antibiotics/AMR?
    a. Awareness/advocacy?

**Funding or fund-raising:**

10. Can you provide details on how your organization would address any financial constraints or issues that would affect the access of an antibiotic or drug?
11. Can you detail any funding models or mechanisms your organization employs for addressing AMR and providing support for antibiotic-related initiatives?
    a. This could include grants, partnerships & collaborations, government grants, public and private sector engagement, subscription or pooled-procurement models

**1f. Interview Guide for Health Technology Assessors**

**Baseline questions:**

1. How many years have you been working in this field and organization?
2. What is your role with regards to antibiotic assessment?
3. Could you elaborate your team, their roles and involvement in the HTA process for antibiotics?
4. How does the HTA team complement the national regulatory agency?

**HTA Review:**

1. Do you review health technologies (antibiotics) purely on a submission basis from a client or do you proactively try to review certain technologies (antibiotics)?
2. Do you review all health products including antibiotics that are looking to enter your country? If not, please explain the process, e.g. regulatory agency suggests products for review.
3. What's your criteria for assessing antibiotics? Do you have certain things to look out for assessment, e.g. cost, patients, efficacy?
    a. And within those categories what is reviewed?
    b. Can you detail the process of this assessment step by step?
4. Can you explain the process for assessing antibiotics?
5. Do you use or reference international HTA guidelines to assist your assessment, e.g National Institute for Health and Care Excellence (NICE) in the United Kingdom or the Agency for Healthcare Research and Quality (AHRQ) in the United States.
6. Are there any significant milestones or accreditations that impact your HTA assessment, e.g. clinical trials, approval by regulatory agencies overseas FDA, EMA.

**Training:**

7. What are the kinds of training and education of the product provided by HTA?
    a. How often are they conducted?
    b. What is the feedback from these trainings and subsequent follow up?

**Awareness:**

8. Does your HTA agency collect information from the public about antibiotics and AMR?
    a. If yes, to what extent do you think the public in your country is informed about antibiotics and AMR issues?
    b. If no, if that is not your domain area, who in your country would be responsible for this?
9. Are there any strategies or efforts from the governments to provide adequate information or education to the public about antibiotics and AMR?
10. Would you like to add anything else relevant that we have missed?

**1g. Interview Guide for Patient and Patient Advocacy Groups**

**Baseline questions:**

1. Could you give us a brief self-introduction, like how many years have you been a patient advocate; what is your current position?
   OR
   Could you give us a brief self-introduction, like how many years have you been battling AMR?
2. What does a [*current position*] do within the organisation? How many active patients does the organisation has? What kind of patients does the organisation usually meet / their common characteristics? Is the organisation representing patients internationally – which countries?
3. Have you met any patients who faced AMR infections? What are their common concerns when dealing with their AMR health issues?

**Appropriate use of antibiotics and antibiotic resistance:**

1. Have you heard of antibiotic resistance? If yes, what do you know about it?
2. How much do you think patients around you know and understand the threat of antibiotic resistance? (prompt: are they aware that antibiotics should not be used in viral infections like common colds? knowledge of drug-resistant infections in hospital settings)
3. How concerned do you think patients are about antibiotic resistance for i. themselves; ii. in the community?
4. How closely do you think patients around you follow doctors' instruction when taking antibiotics, such as completing the whole course and not missing doses?
5. Can you think of a time when someone you know didn't take antibiotics as prescribed by their doctor? Why do you think that happened?

**Source of information and education:**

1. Where do you or your patients around you usually get information about antibiotics (safety, benefits, usage) and antibiotic resistance? (e.g., from healthcare professionals, the internet, friends)
2. What kinds of information do you or your patients receive on antibiotic resistance, for example completing courses of antibiotics, drug-resistant infections, antibiotic usage in animals.
3. How often do you or do you think patients around you would receive information and education about antibiotics and antibiotic resistance?
4. How helpful do you or they find this information about antibiotics?
5. How much do you or patients around you trust the information provided by
   a. Governments and health authority
   b. Mass media
   c. Social media
   d. Healthcare professionals
   e. Pharmaceutical companies
6. What role do you think governments and health authorities should play in addressing the issue of antibiotic resistance?
7. To what extent do patients around you receive enough information and education about antibiotics and antibiotic resistance from these sources? If not, what do you think are the reasons for the lack of information?

**Awareness of the availability of antibiotics:**

1. Where do you or patients around you usually get antibiotics from?
2. How aware are the patients around you that newer antibiotics are needed to address antibiotic resistance? If they are aware, how concerned are they about the lack of access to newer antibiotics for antibiotic-resistance infections?
3. Who do you think are responsible for ensuring that patients have adequate access to newer antibiotics?
4. What do the patients think is their role in managing this issue of antibiotic resistance?
5. Have you seen changes in patient perceptions in terms of knowledge- seeking, awareness of antibiotics and resistance? e.g. they may be more cautious of antibiotics or seeking care.
6. Would you like to add anything else relevant that we have missed?

## 2. Results

### Table S1. Themes, sub-themes with representative quotes

| Themes | Sub-themes | Quotes |
|---|---|---|
| Misaligned mindset | Differing urgency in HIC vs LMIC | “We are at this slow boiling point, where there isn't that much urgency, but yet, you can see that it is clearly affecting how healthcare is practiced and affecting human outcomes in many patients, right? I have only seen this happen on two occasions for infectious diseases, one of course, for COVID (Coronavirus Disease). And the other for HIV (Human Immunodeficiency Virus) in the 1980s and 1990s and drugs became available. For AMR, we have not reached this point. Part of the problem is, and I consider this a problem, is that there are always other options. Even if these options are inferior, they are still available. If you have zero drugs or zero options, then yes, it becomes a major problem.” - POL1 (HIC) |
| | | “I think it (AMR) is a problem. But again, it's a niche problem. And I think, we run the risk of over-dramatizing it. So, because most infections actually can be treated, and a lot of times people with antibiotic resistant bacteria isolated are not infected, that they are colonized with these bacteria.” - HCP1 (HIC) |
| | Uncertainties over efficacy of newer antibiotics | “The main thing is that these drugs don't really work… in other words, you're asking me about access to a drug which doesn't work. And a drug which may not work most of the time… I mean, frankly, ceftazidime-avibactam has never been shown superior to Polymyxin or to any of the other comparator agents in the clinical trial. All the trials have been done as the non-inferiority trial.” - HCP1 (HIC) |
| | | “For us, deciding whether to pay for a new antibiotic really comes down to comparative evidence. When we look at newer antibiotics, the data comparing them to what they're meant to replace often just isn't there. So, you can't really conclude that the new drug is better than the old one. And even more than that - how do you justify the price? Sometimes these new antibiotics cost several hundred percent more than older ones. As you rightly mentioned, they're super expensive - way overpriced. So, whether that's a fair price to pay, we would need to evaluate it carefully.” - HTA1 (HIC) |
| | Creeping normality diminish perceived urgency | “If they know the organism is really resistant, they use polymyxins - but not all hospitals (in LMIC) have access to them. So, basically there's nothing to use.” - HTA2 (HIC) |
| | Limited patient advocacy | “Not enough, not enough (information about AMR is being shared with patients). Because it might be at a level where a bunch of doctors are speaking at a seminar. But a total of 100 patients attend while there are 3000 patients affected...so not enough information is being shared. And that's clinicians speaking at that one platform. And that's it." - PAG2 (HIC) |
| Competing priorities and reactive approaches | Focusing stewardship towards optimising existing antibiotics | “The issue is that agents like cefiderocol, which can cover most of the gram negatives or ceftazidime-avibactam, which could again work in a similar way, are not yet widely available. The second thing is the significant issue of cost. For example, if you take ceftazidime-avibactam, till Pfizer had the patent which is about a little more than a year or two, ceftazidime-avibactam was quite expensive for regular use for all patients. So, secondly, there is an issue of cost.” - HCP 7 (LMIC) |
| | | “I don't think that the priority in most of this country is about access to new antibiotics. I think the priority is about ensuring that the existing antibiotics are adequately used.” - GHA7 |
| | Short-term workarounds undermine long-term efforts | “Yes, so for example, there were about I think, 90 patients who requested access for this drug across xx country. And finally, the regulatory approvals, the number of patients that use the drug, the number of clinicians that got access finally is approved. Because |

| | | |
|---|---|---|
| | | these are very sick patients, quite a few of them died, when you ask for approval itself, and four of them are from xx hospital." - HCP 7 (LMIC) |
| | | "They (public hospitals) procure the medication at the beginning of the year. But if you use it (ceftazidime-avibactam) up, then that's it. That's the end of the story. Because you are going to go back to polymyxin B-based therapy or polymyxin-based therapy for this patient." - HCP6 (LMIC) |
| | Infection Prevention and Control (IPC) more attainable than antibiotic access | "I think IPC is not prioritised at a high level. I think that when we talk about the AMR of high-income countries, they always put a new antibiotic at the top. 'Where are the new antibiotics? Put more funding into the analytics.' Which is okay, which is good. You should put something about that, but you should not forget IPC and save lives that you can save now…if it's Cambodia, Laos, Africa, I would push sanitation and public water as priority." - POL10 (LMIC) |
| Lack of systemic investment | No unified dataset to inform policies | "When you do a case study, you need to compare with a reference, it's like a checklist as well. In HTA, we don't have that guideline or reference case for antibiotic evaluation. And the lack of this reference case of guidelines is not because people do not see the importance but because it is really difficult. Many teams have tried to address this by developing recommendations, guidelines, and references, but all have failed so far." HTA3 (LMIC) |
| | | "…but it's also how much robustness or weight we can give to such data. Yeah, so I think it (evidence) has to be case by case, assessed by what kind of data that is being received" - HTA1 (HIC) |
| | | "I think that cost is the problem (to getting essential antibiotics). But the real problem is the lack of research in this field. So if we have good evidence to prove to the policymaker that it will work, we can order this (cefiderocol and ceftazidime-avibactam) drug. Every year we had to make a budget plan to the government and the budget plan based on the need and based on the number of the different types of the drugs that we use. We don't have much research evidence to show that we really need this drug in the community or in the hospital." - HCP8 (LMIC) |
| | | "If the demand is not sufficient, then the investor will face difficulties in the future. So it's really difficult to invest huge money in huge facilities. If they really want to invest money in these facilities, they need to understand how much or how much products the world will need, and it should be constant, but it's very difficult to predict." - IND3 |
| | | "So far, we use only the international partner budget, and have not used the national budget yet. But this year and next year, I proposed an allocation of the national budget to support the AMR… We do not know (the outcome) yet. On the way to the negotiation." - POL9 (LMIC) |
| | Limited capacity to conduct clinical trials | "I think the biggest challenge we have for small companies is getting a meaningful clinical trial done at a reasonable cost. If I run a trial through a CRO (Contract Research Organization) at, let's say 200 patients in an Intensive Care Unit. I'm sure this was a $50 million trial, right, with all the CRO costs and everything behind it, and now you want to have that for 8-10, different drugs, you're looking at a half a billion and these are abbreviated clinical development pathways." - IND3 |
| | | "It would be great to have some kind of alternative pathways for doing clinical trials in a more cost-efficient manner. And I think there's a benefit in being able to do them in certain countries where burden is quite high and patients are easier to enrol from that perspective." - GHA6 |
| | | "We generally look at published trials from the companies, because any local data, like real world evidence, is more |

| | | |
|---|---|---|
| | | supplementary nature. We focus on whatever published evidence is out there, because they are typically sponsored by the companies manufacturing and that's the best available evidence we have. Some of these local data on antibiotic resistance patterns and all, we will take into consideration when we also look at establishing the clinical need, the place in therapy for the treatment, but they are not used to establish the comparative evidence." - HTA1 (HIC) |
| | Disconnect between public health goals and industry incentives | "Schemes that improve predictability are really important. Pooled procurement agencies, e.g. Global Fund and GDF (Global Drug Facility) work because supply and access is standardised, despite there still being a need for local registration. … I think the other problem is just the uncertainty of the market, which is, maybe there's not great demand forecasting. It's kind of the equivalent in the antibiotic space and so a company doesn't know what the demand will be this year, next year, or 10 years from now. It's going to look very different and not just because the science changes, but because a country or a hospital itself doesn't necessarily know." - GHA6 |
| | | "So the biggest casualty of our poor antibiotic sustainability is going to be the research teams, discovery teams, and once they are gone, your source of the new antibiotic itself is gone. There will be no discussion of access." - IND5 |
| | | "The driving force for companies or industries is to go first to the FDA (Food and Drug Administration), because the access to this is more straightforward than in Europe. And we've seen that recently, some new antibiotics that have been approved have never been licensed in Europe. And that's simply due to the cost of going to each one of these countries separately. …75% in the market, I would need three approvals, basically, whereas then to gather the other 25% you would have to go through each country separately. That's the driving force we need to get as a company. We need to show the investors, to everyone, that we're going to the current markets. But this doesn't necessarily mean that this is where the medical or unmet medical need really is. And that is a bit of the conundrum that we're living in — that we need to go to the market, yet the medical need is often elsewhere." - IND4 |
| | Weak system infrastructure impedes approval and uptake | "Unfortunately, the approval of medicines, we have a huge backlog. From 2019 to 2024, those four years, there's a backlog. We have a reason for the backlog, so one is the shortage of staff and the poor expertise of the regulatory staff. So, because of the running out of staff, I'm doing the registration of the medical devices, of the medicines, and of cosmetics." - REG1 (LMIC) |
| | | "So how the relationship between the countries is one of the points for us, that is true. And second one is about the IP (Intellectual Property) in the country. But if we try to enter a developing country, some countries are not good at protecting IP. In this case, we cannot enter. We cannot." - IND2 |
| | | "But then, until the official either FDA (Food and Drugs Administration) or CLSI (Clinical and Laboratory Standards Institute) or EUCAST (European Committee on Antimicrobial Susceptibility Testing) breakpoints and a standardised methodology, it's hard to steward or decide who is appropriate for this antibiotic. For example, ceftazidime-avibactam with aztreonam, there's no breakpoint yet, unless you infer from aztreonam breakpoints. And there's also no standard method currently, so there's no device. There's no CLSI or recognised method or breakpoint." - HCP2 (HIC) |
| | | "There are no good ways to sort of suspect who has carbapenem resistant infection. And if the diagnostic setup is not very good, your diagnosis and identifying these patients itself is a problem. So that brings in a huge amount of challenge for a public hospital to sort of identify these patients." - HCP7 (LMIC) |

| | | |
|---|---|---|
| | | “You have a very limited stock, sometimes you also don't know which patient you should give (the medicine to), you see? In the end, you don't use it because you feel like you do not know - which patient should I start? In the end, you will go back to polymyxin and colistin. Same goes to ceftazidime-avibactam. Six months already in this hospital, but none of the doctors want to start because we have limited stock and they are also confused which patient to give.” - HCP9 (LMIC) |
| | | “If you look at all global literature, from IDSA (Infectious Diseases Society of America) or ECCMID (European Congress of Clinical Microbiology and Infectious Diseases) they would say they would clump Klebsiella and E. coli together. And then say that for Enterobacteriaceae, say, you use ceftazidime-avibactam with confidence, this will be one of the first line drugs to use. But in (a LMIC country) you can't translate that because E. coli will not work most of the time but for Klebsiella it will work phenomenally well. So in (a LMIC country), when you come up with an empirical or even a definitive therapy, waiting for molecular confirmation, you have to use ceftazidime-avibactam plus aztreonam. Then with pseudomonas, your first claim is again, ceftazidime-avibactam, ceftolozane tazobactam, and things like that, which won't work here, because of the MBL (metalo-betalactamase resistance) rates in Pseudomonas. So we need a lot of caution in translating these guidelines across.” - HCP7 (LMIC) |
| | | “We have certain criteria, whether it's high clinical need evidence and then prioritise certain topics. There has never been like a big impetus for us to sort of look at antibiotics all this time, in part because a lot of antibiotics that are used in the inpatient setting, there's (insurance) coverage for inpatients. ... As we shared, there's only one team looking at all the topics, then it's about prioritizing which topics, because there's only three meetings per year and only a fixed number of topics each year.” - HTA1 (HIC) |
| Absence of coordinated leadership | Regulatory fragmentation | “I think when we spoke to a lot of the regulatory agencies, they feel typical things like understaffed, underfunded, they may start to band together, but I think there needs to be like champions within to drive that from an AMR focus because they're not going to have a specific AMR regulatory person - they do general approvals.” - GHA3 |
| | | “Harmonisation is helpful, I think, from the point of each new market launch being smaller incremental works rather than having to start from scratch. From the perspective of everyone, there has to be a common understanding of what’s required to prove that this product is effective, it’s safe, it’s quality assured etc. That there aren’t different ways of assessing this.” - GHA6 |
| | Siloed stakeholders deter ownership | “There is not a central authority that dictates the AMR policy response. It's usually a conglomerate of different players and actors that try and work together. Even in the United States, even the United Kingdom, you have more emphasis on this. It belongs to a lot of stakeholders, and I don't know whether there's one person who is full, like, accountable for these problems.” - POL2 (HIC) |
| | | “I think, ultimately, it (access) has to be a priority of the Ministry of Health. We help develop public or private health programs which ultimately will sustain after we (GHA) intervene, they tend to be owned or registered by the government.” - GHA1 |
| | Gap unaddressed by technical and philanthropic actors | “We should keep it in mind that those (encouragement to novel conceptual interventions such as vaccines, phages, diagnostics by Combating Antibiotic-Resistant Bacteria Biopharmaceutical Accelerator [CARB-X]) will not yield you a viable product which can be used in the patient, where you can save the life of today's patients or tomorrow's patients. … So the question is, are our funding agencies, supporting agencies maintaining a balance between supporting a product which will come 10-15 years down the line, or whether they are supporting a project which can become a lifesaving project in just the coming three or four years.” - IND5 |

| | | |
|---|---|---|
| | | “And so, we are dependent on the (xx GHA) because their goal would be to help bring companies forward and bring new drugs to the market, right? That should be their goal. So we are all somewhat looking for support from them. But keep in mind, that the support is generally very small. They haven’t invested very much in any company, and so it’s not a lifesaver.” - IND4 |

Abbreviations: HIC, high-income country; LMIC, low- and middle-income country; AMR, antimicrobial resistance; POL, policy maker; HCP, healthcare professional; HTA, health technology assessor; PAG, patient advocacy group; GHA, global health actor; IND, industry experts; REG, regulators.

**Table S2: COREQ statement**

| Item number | Guide questions/description | Reported (page/details) |
|---|---|---|
| **Domain 1: Research team and reflexivity** | | |
| *Personal characteristics* | | |
| 1. Inter viewer/facilitator | Which author/s conducted the interview or focus group? | Pages 31 to 32, lines 690 to 698 |
| 2. Credentials | What were the researcher's credentials (e.g. PhD, MD)? | Author's list |
| 3. Occupation | What was their occupation at the time of the study? | Page 10, line 185 |
| 4. Gender | Was the researcher male or female? | Author's list |
| 5. Experience and training | What experience or training did the researcher have? | Page 10, line 186 to 187 |
| *Relationship with participants* | | *Relationship with participants* |
| 6. Relationship established | Was a relationship established prior to study commencement? | Page 10, line 167 to168 |
| 7. Participant knowledge of the interviewer | What did the participants know about the researcher? e.g. personal goals, reasons for doing the research | Page 10, line 178 |
| 8. Interviewer characteristics | What characteristics were reported about the inter viewer/facilitator? e.g. bias, assumptions, reasons and interests in the research topic | Page 10, line 185 |
| **Domain 2: Study design** | | Domain 2: Study design |
| *Theoretical framework* | | *Theoretical framework* |
| 9. Methodological orientation and theory | What methodological orientation was stated to underpin the study? e.g. grounded theory, discourse analysis, ethnography, phenomenology, content analysis | Page 10, line 210-11 |
| *Participant selection* | | *Participant selection* |
| 10. Sampling | How were participants selected? e.g. purposive, convenience, consecutive, snowball | Page 9, line 169 |
| 11. Method of approach | How were participants approached? e.g. face-to-face, telephone, mail, email | Page 9, line 169 |
| 12. Sample size | How many participants were in the study? | Page 11, line 213 |

| | | |
|---|---|---|
| 13. Non-participation | How many people refused to participate or dropped out? Reasons? | Page 11, line 213 to 215 |
| *Setting* | | *Setting* |
| 14. Setting of data collection | Where was the data collected? e.g. home, clinic, workplace | Page 10, line 191 |
| 15. Presence of non-participants | Was anyone else present besides the participants and researchers? | NA |
| 16. Description of sample | What are the important characteristics of the sample? e.g. demographic data, date | Page 11, lines 212 to 215 |
| *Data collection* | | *Data collection* |
| 17. Interview guide | Were questions, prompts, guides provided by the authors? Was it pilot tested? | Page 10, line 186 |
| 18. Repeat interviews | Were repeat inter views carried out? If yes, how many? | NA |
| 19. Audio/visual recording | Did the research use audio or visual recording to collect the data? | Page 10, line 192 |
| 20. Field notes | Were field notes made during and/or after the inter view or focus group? | Page 10, line 188 |
| 21. Duration | What was the duration of the inter views or focus group? | Page 10, line 192 |
| 22. Data saturation | Was data saturation discussed? | Page 11, line 207 |
| 23. Transcripts returned | Were transcripts returned to participants for comment and/or correction? | Page 11, line 207 to 210 |
| **Domain 3: Analysis and findings** | | Domain 3: Analysis and findings |
| *Data analysis* | | *Data analysis* |
| 24. Number of data coders | How many data coders coded the data? | Page 10, line 198 to 199 |
| 25. Description of the coding tree | Did authors provide a description of the coding tree? | Pages 10 to 11, lines 200 to 201 |
| 26. Derivation of themes | Were themes identified in advance or derived from the data? | Page 11, lines 202 to 205 |
| 27. Software | What software, if applicable, was used to manage the data? | Page 10, lines 196 to 197 |
| 28. Participant checking | Did participants provide feedback on the findings? | Page 32, line 692 to 694 |
| *Reporting* | | |
| 29. Quotations presented | Were participant quotations presented to illustrate the themes/findings? Was each quotation identified? e.g. participant number | Yes |

| | | |
|---|---|---|
| 30. Data and findings consistent | Was there consistency between the data presented and the findings? | Yes |
| 31. Clarity of major themes | Were major themes clearly presented in the findings? | Yes |
| 32. Clarity of minor themes | Is there a description of diverse cases or discussion of minor themes? | Yes |
