# Supplementary material for: Barriers to accessing newer antibiotics in countries with high burden of bacterial antimicrobial resistant infections—a qualitative study
Source: Lancet Reg Health West Pac. 2026 Jul 16;72:101917. doi: 10.1016/j.lanwpc.2026.101917 (PMC13400380; doi:10.1016/j.lanwpc.2026.101917)
Supplement: Case boxes_ABxAccess [file mmc2.docx]

**Case Study Box 1: Ceftazidime-avibactam**

*Early phase clinical trials*

Ceftazidime-avibactam (CZA) is a broad-spectrum third-generation cephalosporin designed for carbapenem-resistant Gram-negative bacterial (GNB) infections.^1^ Ceftazidime works by binding to penicillin-binding proteins (PBPs), primarily the PBP-3, in Gram-negative bacteria, while avibactam acts by forming a covalent and reversible bond to serine β -lactamases to protect ceftazidime from hydrolysis.^2^ *In vitro* studies have demonstrated the efficacy of CZA against extended-spectrum beta-lactamases (ESBL)-producing *Klebsiella pneumoniae,* carbapenemase (KPC)-producing Enterobacterales, and *Pseudomonas aeruginosa*.^3,4^

Phase three clinical trials have shown CZA to be non-inferior to carbapenem and other standard antibiotic comparators for treating multidrug-resistant GNB infections.^5^ The REPROVE trial evaluated CZA against meropenem for nosocomial pneumonia^6^ while the REPRISE trial compared CZA with best available therapy for complicated urinary tract (cUTI) and intra-abdominal infections (cIAI) caused by ceftazidime-resistant Enterobacterales and *Pseudomonas aeruginosa*.^7^ Both these trials demonstrated that CZA is an effective and safe alternative to carbapenems for MDR GNB infections. Data analysis from a pooled population study of different CZA clinical trials confirmed its safety profile as comparable to ceftazidime monotherapy.^8^ Similarly, in a systematic review and meta-analysis of efficacy and safety of CZA against CRE infections, patients treated with CZA had lesser mortality than comparators, thereby supporting CZA’s inclusion in treatment guidelines for CRE infections.^9^ The 2024 IDSA and ESCMID guidelines recommended its use in carbapenem-resistant GNB infections, despite the absence of trials that were powered to assess CZA’s superiority for carbapenem-resistant infections.^5,10^

*Regulatory approval and manufacturing timeline*

CZA is commercialised by Allergan in the United States and Canada, and by Pfizer in other global markets.^11^ The drug was first approved by the US FDA in February 2015 for use in adult patients with cIAI.^11,12^ In the same year, FDA designated CZA as Qualified Infectious Disease Product (QIDP).^13^ The QIDP status prioritised CZA’s review for new indications and formulations and fast-tracked its subsequent approvals, thus furthering its access by extending its market exclusivity. In 2016, the European Union granted approval to CZA for serious infections, including cUTI, cIAI, hospital-acquired bacterial pneumonia (HABP), and ventilator-associated bacterial pneumonia (VABP).^14^ In 2019, US FDA approved drug labelling extension to paediatric patients, aged three months and older, with cUTI and cIAIs.^15^ By 2018, CZA had been granted approval in various countries, including the EU, Russia, Argentina, Brazil, Colombia, China, and India.^16^

*Challenges in access*

Despite the successful registration of CZA in a few major countries, since 2021, CZA has been approved in only 20 of 102 LMICs.^17^ This underrepresentation underscores the difficulties in commercialising newer antibiotics as regulatory delays in LMICs can span up to seven years.^18^

Even after CZA was granted market exclusivity following its approval, this exclusivity was brief in some countries.^19^ In India, within five years of its launch, CZA lost its exclusivity as multiple companies began manufacturing generic versions of the drug. This shift was partly caused by a lack of emphasis on data exclusivity and the passing of the Indian Patents Act, which allowed manufacturers to produce generic versions to ensure drug affordability.^20^ The loss of market exclusivity might prove a deterrent to pharmaceutical companies investing in Research and Development of new antibiotics.

Due to limited alternative therapies and the increasing burden of MDR-GNB infections, CZA is increasingly being used to treat them.^21^ As CZA resistance increases in regions with widespread use, its clinical effectiveness diminishes due to emerging resistance patterns.^22^ One key resistance mechanism involves mutations in the PBP3 protein where CZA normally binds to inhibit cell wall synthesis. These mutations impair binding of the drug, thereby reducing its effectiveness.^23^ While CZA is effective against bacteria that produce OXA-48 enzymes to break down carbapenems, this is not applicable to other resistance types such as NDM- producing bacteria, which are more prevalent in parts of South and Southeast Asia, thereby limiting CZA’s utility in these settings.^24,25^ A systematic review revealed that Asia has higher rates of CZA resistance compared to other regions such as Africa, Europe, South America, and North America.^26^ In some ICU settings in China, CZA resistance is often found in carbapenem-resistant *K. pneumonia* and *P. aeruginosa*.^27^ In Italy, CZA resistance in *P. aeruginosa* varies by region, as some areas reported links to MBL and ESBL enzymes, while other regions identified mutation strains like ST111 and ST253.^28^

Economic evaluations of CZA across different economic settings have created varied assessments around its cost-effectiveness, therefore influencing pricing, formulary inclusion, and reimbursement strategies. A cost-effective study conducted in a regional Chinese health setting noted a reduction in hospitalisation costs and improvement of health outcomes, thereby generally assessing the drug as cost-effective.^29^ In Brazil, despite the drug’s high cost, it was seen as more cost-effective than Polymyxin, considering factors such as Quality-Adjusted Life Years (QALY), willingness to pay threshold (WTP), and Incremental Cost-Effectiveness Ratio (ICER).^30^ In a budget impact analysis of CZA in Singapore’s healthcare setting, the introduction of CZA in treating patients with limited treatment options had a minimal impact on the overall healthcare budget.^31^ However, conducting health economic evaluations of newer antibiotics such as CZA in LMIC settings can be challenging as high drug cost, high AMR burden and low capacity for generating quality data can complicate cost-effective analyses.^32^

In conclusion, barriers to CZA access, such as late regulatory approval in LMICs compared to HICs, poor returns on investment for pharmaceutical companies to develop new antibiotics, emerging resistance due to widespread use, and varying cost-effective evaluations, highlight the urgent need for a cross-disciplinary approach to improve access to newer antibiotics.

**CASE Study Box 2: Cefiderocol**

*Early phase clinical trials*

Cefiderocol is a siderophore cephalosporin targeting GNB infection, especially those with carbapenem resistance.^33^ It functions by binding to extracellular iron, allowing it to be transported across cell membranes, thereby inhibiting bacterial cell wall synthesis.^34^ Its unique mechanism enhances the drug’s effectiveness against multidrug-resistant, especially carbapenem-resistant (CR) GNB infections.^35^

Later phase trials, namely the Phase III APEKS-NP and Phase II APEKS-UTI, show that cefiderocol is non-inferior to the best available treatment. APEKS-NP was a double-blind, phase III, non-inferiority, randomised trial comparing the efficacy and safety of cefiderocol to extended meropenem infusion against nosocomial pneumonia.^36^ APEKS-UTI was a phase II, double-blind, noninferiority trial assessing cefiderocol and imipenem-cilastatin in treating cUTI.^37^ CREDIBLE-CR investigated the efficacy of cefiderocol compared to best available therapy against infections caused by CR-GNB.^38^ The study found that cefiderocol has similar efficacy compared to the best available therapy; however, with a notably higher mortality among patients with *Acinetobacter* spp. infections.

The IDSA 2024 guidelines suggested cefiderocol as an option for treating pyelonephritis and cUTI caused by CR-GNB, but not for infections associated with a non-urinary source.^5^ As there was no data showing the effectiveness of cefiderocol against KPC-producing Enterobacterales, cefiderocol was only recommended as an alternative option against CRE, Carbapenem-resistant *Acinetobacter baumaniii*, and ESBL producing Enterobacterales. Given the limited clinical evidence, the 2022 ESCMID guidelines recommend cefiderocol only for patients with few treatment options.^10^

*Regulatory approval and manufacturing timeline*

Cefiderocol is developed and marketed by the pharmaceutical company Shionogi.^39^ The drug received its first approval from the US FDA in October 2019 for the treatment of adult cUTI and was designated as QIDP under the GAIN Act, making it eligible for fast track status to expedite the review and approval of the drug.^40^ This was followed by the FDA setting a Prescription Drug User Fee Act (PDUFA) timeline, thereby further accelerating its access. In April 2020, the European Union granted marketing authorisation^41^, and later that same year, the US FDA approved cefiderocol for the treatment of HABP and VABP cases.^42^ Since the FDA approval in 2019, approval has been provided by Japan (2022), Taiwan (2024) and South Korea (2025).^43-45^ However, expansion to LMIC Asian countries remains limited, and they are only able to access cefiderocol through compassionate use.^46^

*Challenges in access*

Due to the unique chemical properties of cefiderocol, the manufacturing and scale up of the drug present significant challenges. Production requires dedicated sterile facilities to prevent cross-contamination, with a specialised facility for lyophilization process. Maintaining controlled conditions — particularly low humidity and low temperature, are essential to ensuring product stability and drug efficacy.^47^ There are significant delays in procuring this custom manufacturing equipment as they are supplied by only one or two global suppliers, which hinders the manufacturing scale-up efforts. Because of the complex manufacturing process of cefiderocol and its initial focus on commercialisation in high-income settings, access in low-resource settings was significantly affected.^48^

In June 2022, a landmark license agreement was established between Shionogi, the Global Antibiotic Research and Development Partnership (GARDP) and Clinton Health Access Initiative (CHAI) to expand cefiderocol access in 135 countries.^49^ This collaborative agreement aimed to improve cefiderocol access through sub-licensing, particularly in countries with delayed access. This collaborative agreement was established to facilitate rollout efforts, including ensuring quality assurance, stewardship, policy guidance, and market shaping. Cefiderocol’s sublicensing for manufacturing was awarded to Orchid Pharma, an India-based company, specifically to build a specific facility for the active pharmaceutical ingredient (API) – a cephalosporin core with siderophore, and the finished dosage form (FDF). This facility handles the in-vial lyophilization of the drug, which is a crucial step in manufacturing and scale up. The technology transfer by Shionogi and its facilitation by GARDP were designed to expand access and to serve as a template for future antibiotic access collaborations.^50^

The assimilation of the drug into clinical practice, specifically in susceptibility testing and drug administration, also presents complexities. Since cefiderocol relies on bacterial iron transport under low-iron conditions for bacterial cell entry, accurate susceptibility testing requires the use of iron-depleted media to replicate in vivo activity.^51^ This requirement complicates the integration of the drug in conventional antimicrobial susceptibility testing (AST) platforms, where standard media formulations are used.^52^ While there are other developed commercial testing available for cefiderocol, this is still met with questionable accuracy and is not widely available. Furthermore, cefiderocol is administered via a prolonged, 3-hour infusion requiring a dedicated port that can allow simultaneous infusion of compatible drugs and flushing without disconnecting to the primary IV site making cefiderocol a specialised drug to administer.^53^

Recent data show reduced susceptibility to cefiderocol in metallo-beta-lactamase (MBL) producing strains, particularly harbouring NDM variants.^54^ This warrants caution in using the drug against NDM variants, emphasising the need for close monitoring of the patient’s clinical trajectory. The growing resistance of cefiderocol and its clinical safety findings may raise concerns about the drug’s long-term efficacy. Notably, findings from phase III trials such as CREDIBLE-CR observed a higher all-cause mortality rate in patients with cefiderocol compared to best available therapy, especially in patients with *A. baumanii* infections.^38^ These outcomes underscore the need for the cautious use of cefiderocol to only within its indicated guidelines.

The case of cefiderocol access underscores the multifaceted barriers that can delay equitable access to newer antibiotics. From complex manufacturing constraints, clinical safety issues, challenges in clinical assimilation, and emerging resistance. Acknowledging these barriers is essential to create innovative approaches to antibiotic access.

**REFERENCES:**

1. Shirley M. Ceftazidime-Avibactam: A Review in the Treatment of Serious Gram-Negative Bacterial Infections. *Drugs (New York, NY)* 2018; **78**(6): 675-92.

2. Ehmann DE, Jahić H, Ross PL, et al. Avibactam is a covalent, reversible, non–β-lactam β-lactamase inhibitor. *Proceedings of the National Academy of Sciences* 2012; **109**(29): 11663-8.

3. Isler B, Vatansever C, Özer B, et al. Comparison of ceftazidime-avibactam susceptibility testing methods against OXA-48-like carrying Klebsiella blood stream isolates. *Diagnostic Microbiology and Infectious Disease* 2022; **104**(1): 115745.

4. Lemos-Luengas EV, Renteria-Valoyes S, Cardenas-Isaza P, Ramos-Castaneda JA. In vitro activity of ceftazidime/avibactam against Gram-negative strains in Colombia 2014–2018. *Journal of Global Antimicrobial Resistance* 2022; **29**: 141-6.

5. Tamma PD, Heil EL, Justo JA, Mathers AJ, Satlin MJ, Bonomo RA. Infectious Diseases Society of America 2024 guidance on the treatment of antimicrobial-resistant gram-negative infections. *Clinical infectious diseases* 2024: ciae403.

6. Torres A, Zhong N, Pachl J, et al. Ceftazidime-avibactam versus meropenem in nosocomial pneumonia, including ventilator-associated pneumonia (REPROVE): a randomised, double-blind, phase 3 non-inferiority trial. *The Lancet Infectious Diseases* 2018; **18**(3): 285-95.

7. Carmeli Y, Armstrong J, Laud PJ, et al. Ceftazidime-avibactam or best available therapy in patients with ceftazidime-resistant Enterobacteriaceae and Pseudomonas aeruginosa complicated urinary tract infections or complicated intra-abdominal infections (REPRISE): a randomised, pathogen-directed, phase 3 study. *The Lancet Infectious Diseases* 2016; **16**(6): 661-73.

8. Cheng K, Newell P, Chow JW, et al. Safety profile of ceftazidime–avibactam: pooled data from the adult phase II and phase III clinical trial programme. *Drug Safety* 2020; **43**(8): 751-66.

9. Chen Y, Huang H-B, Peng J-M, Weng L, Du B. Efficacy and safety of ceftazidime-avibactam for the treatment of carbapenem-resistant Enterobacterales bloodstream infection: a systematic review and meta-analysis. *Microbiology Spectrum* 2022; **10**(2): e02603-21.

10. Paul M, Carrara E, Retamar P, et al. European Society of Clinical Microbiology and Infectious Diseases (ESCMID) guidelines for the treatment of infections caused by multidrug-resistant Gram-negative bacilli (endorsed by European society of intensive care medicine). *Clinical Microbiology and Infection* 2022; **28**(4): 521-47.

11. AbbVie News Center. FDA Approves AVYCAZ® (ceftazidime and avibactam) for the Treatment of Patients with Hospital-Acquired Bacterial Pneumonia and Ventilator-Associated Bacterial Pneumonia. Feb 1, 2018. <https://news.abbvie.com/2018-02-01-FDA-Approves-AVYCAZ-R-ceftazidime-and-avibactam-for-the-Treatment-of-Patients-with-Hospital-Acquired-Bacterial-Pneumonia-and-Ventilator-Associated-Bacterial-Pneumonia> (accessed Oct 1, 2025).

12. Mosley JF, Smith LL, Parke CK, Brown JA, Wilson AL, Gibbs LV. Ceftazidime-avibactam (Avycaz): for the treatment of complicated intra-abdominal and urinary tract infections. *Pharmacy and Therapeutics* 2016; **41**(8): 479.

13. Food Drug Administration (FDA). Qualified Infectious Disease Product Designation—Questions and Answers Guidance for Industry*.* 2021. https://www.fda.gov/regulatory-information/search-fda-guidance-documents/qualified-infectious-disease-product-designation-questions-and-answers (accessed Oct 1, 2025).

14. Astrazenaca. New antibiotic Zavicefta approved in the European Union for patients with serious bacterial infections. June 28, 2016. https://www.fda.gov/regulatory-information/search-fda-guidance-documents/qualified-infectious-disease-product-designation-questions-and-answers (accessed Oct 1, 2025).

15. PR Newswire. Allergan Announces FDA Approval of AVYCAZ® (ceftazidime and avibactam) for Pediatric Patients. Mar 18, 2019. https://www.prnewswire.com/news-releases/allergan-announces-fda-approval-of-avycaz-ceftazidime-and-avibactam-for-pediatric-patients-300813714.html (accessed Oct 1, 2025).

16. MIMS Singapore. A closer look at ceftazidime-avibactam in real-world settings. Nov 7, 2023. https://www.mims.com/singapore/news-updates/topic/a-closer-look-at-ceftazidime-avibactam-in-real-world-settings (accessed Oct 1, 2025).

17. Access to Medicine Foundation. Pfizer and Viatris use registration to expand availability of on-patent antibiotics in low- and middle-income countries. Nov 18, 2021. https://accesstomedicinefoundation.org/resource/pfizer-and-viatris-use-registration-to-expand-availability-of-on-patent-antibiotics-in-low-and-middle-income-countries (accessed Oct 1, 2025).

18. Ahonkhai V, Martins SF, Portet A, Lumpkin M, Hartman D. Speeding access to vaccines and medicines in low-and middle-income countries: a case for change and a framework for optimized product market authorization. *PloS one* 2016; **11**(11): e0166515.

19. Veeraraghavan B, Bakthavatchalam YD, Sahni RD, Malhotra S, Bansal N, Walia K. Loss of exclusivity of ceftazidime/avibactam in low-and middle-income countries: a test for antibiotic stewardship practice. *The Lancet Regional Health-Southeast Asia* 2023; **15**.

20. The Economic Times. India, UK trade pact does not mandate patent term extensions or data exclusivity. Jul 28, 2025. <https://economictimes.indiatimes.com/news/economy/foreign-trade/india-uk-trade-pact-does-not-mandate-patent-term-extensions-or-data-exclusivity/articleshow/122954451.cms> (accessed Oct 1, 2025).

21. Jorgensen SC, Trinh TD, Zasowski EJ, et al. Real-world experience with ceftazidime-avibactam for multidrug-resistant gram-negative bacterial infections. *Open forum infectious diseases*; 2019: Oxford University Press US; 2019. p. ofz522.

22. Keri VC. Managing carbapenem-resistant gram-negative infections: Challenges in the developing world. Infectious Diseases Society of America (IDSA); Feb 10, 2023. <https://www.idsociety.org/science-speaks-blog/2023/managing-carbapenem-resistant-gram-negative-infections-challenges-in-the-developing-world> (accessed Oct 1, 2025).

23. Periasamy H, Joshi P, Palwe S, Shrivastava R, Bhagwat S, Patel M. High prevalence of Escherichia coli clinical isolates in India harbouring four amino acid inserts in PBP3 adversely impacting activity of aztreonam/avibactam. *Journal of Antimicrobial Chemotherapy* 2020; **75**(6): 1650-1.

24. Sangiorgio G, Calvo M, Stefani S. Aztreonam and avibactam combination therapy for metallo-β-lactamase-producing gram-negative bacteria: A Narrative Review. *Clinical Microbiology and Infection* 2025; **31**(6): 971-8.

25. Wang Y, Wang J, Wang R, Cai Y. Resistance to ceftazidime–avibactam and underlying mechanisms. *Journal of global antimicrobial resistance* 2020; **22**: 18-27.

26. Wang Y, Sholeh M, Yang L, Shakourzadeh MZ, Beig M, Azizian K. Global trends of ceftazidime–avibactam resistance in gram-negative bacteria: systematic review and meta-analysis. *Antimicrobial Resistance & Infection Control* 2025; **14**(1): 10.

27. Zhou J, Chen M, Liang M, et al. Diverse modes of ceftazidime/avibactam resistance acquisition in carbapenem-resistant Klebsiella pneumoniae and Pseudomonas aeruginosa from a Chinese intensive care unit. *Annals of Clinical Microbiology and Antimicrobials* 2025; **24**(1): 35.

28. Valzano F, La Bella G, Lopizzo T, et al. Resistance to ceftazidime–avibactam and other new β-lactams in Pseudomonas aeruginosa clinical isolates: A multi-center surveillance study. *Microbiology Spectrum* 2024; **12**(8): e04266-23.

29. Yang W, Zhen X, Sun X, et al. Estimating the value of new antibiotic treatment strategies in Zhejiang province, China: cost-effectiveness analysis based on a validated dynamic model. *BMJ open* 2024; **14**(8): e086039.

30. Matuoka J, Pachito DV, Piastrelli F, Fehlberg LCC, de Oliveira Junior HA. Economic evaluation of ceftazidime-avibactam vs. polymyxin B for treatment of hospital-acquired and ventilator-associated bacterial pneumonia. *The Brazilian Journal of Infectious Diseases* 2025; **29**(4): 104545.

31. Park D, New J. Budget Impact of Ceftazidime Avibactam (Caz-Avi) for Complicated Intra-Abdominal Infection, Complicated Urinary Tract Infection and Hospital-Acquired Pneumonia in Singapore. *Value in Health* 2019; **22**: S652.

32. Wasan H, Reeta K, Gupta YK. Strategies to improve antibiotic access and a way forward for lower middle-income countries. *Journal of Antimicrobial Chemotherapy* 2024; **79**(1): 1-10.

33. Sato T, Yamawaki K. Cefiderocol: discovery, chemistry, and in vivo profiles of a novel siderophore cephalosporin. *Clinical Infectious Diseases* 2019; **69**(Supplement_7): S538-S43.

34. Abdul‐Mutakabbir JC, Alosaimy S, Morrisette T, Kebriaei R, Rybak MJ. Cefiderocol: a novel Siderophore cephalosporin against multidrug‐resistant gram‐negative pathogens. *Pharmacotherapy: The Journal of Human Pharmacology and Drug Therapy* 2020; **40**(12): 1228-47.

35. Yamano Y. In vitro activity of cefiderocol against a broad range of clinically important Gram-negative bacteria. *Clinical Infectious Diseases* 2019; **69**(Supplement_7): S544-S51.

36. Wunderink RG, Matsunaga Y, Ariyasu M, et al. Cefiderocol versus high-dose, extended-infusion meropenem for the treatment of Gram-negative nosocomial pneumonia (APEKS-NP): a randomised, double-blind, phase 3, non-inferiority trial. *The Lancet Infectious Diseases* 2021; **21**(2): 213-25.

37. Portsmouth S, van Veenhuyzen D, Echols R, et al. Cefiderocol versus imipenem-cilastatin for the treatment of complicated urinary tract infections caused by Gram-negative uropathogens: a phase 2, randomised, double-blind, non-inferiority trial. *The Lancet Infectious Diseases* 2018; **18**(12): 1319-28.

38. Bassetti M, Echols R, Matsunaga Y, et al. Efficacy and safety of cefiderocol or best available therapy for the treatment of serious infections caused by carbapenem-resistant Gram-negative bacteria (CREDIBLE-CR): a randomised, open-label, multicentre, pathogen-focused, descriptive, phase 3 trial. *The Lancet Infectious Diseases* 2021; **21**(2): 226-40.

39. Dobias J, Dénervaud-Tendon V, Poirel L, Nordmann P. Activity of the novel siderophore cephalosporin cefiderocol against multidrug-resistant Gram-negative pathogens. *European Journal of Clinical Microbiology & Infectious Diseases* 2017; **36**(12): 2319-27.

40. Shionogi. U.S. FDA Advisory Committee Recommends Approval of Cefiderocol for Treatment of Complicated Urinary Tract Infections. Oct 16, 2019. https://www.shionogi.com/us/en/news/2019/10/u-s-fda-advisory-committee-recommends-approval-of-cefiderocol-for-treatment-of-complicated-urinary-tract-infections.html (accessed Oct 1, 2025).

41. European Medicines Agency (EMA). Meeting highlights from the Committee for Medicinal Products for Human Use (CHMP) 24-27 February 2020. Feb 28, 2020. https://www.ema.europa.eu/en/news/meeting-highlights-committee-medicinal-products-human-use-chmp-24-27-february-2020 (accessed Oct 1, 2025).

42. Shionogi. Shionogi announces FDA approval of FETROJA® (Cefiderocol) for the treatment of Hospital-Acquired bacterial pneumonia and Ventilator-Associated bacterial pneumonia. Shionogi. Sep 27, 2020. https://www.shionogi.com/us/en/news/2020/9/shionogi-announces-fda-approval-of-fetroja-cefiderocol-for-the-treatment-of-hospital-acquired-bacterial-pneumonia-and-ventilator-associated-bacterial-pneumonia.html (accessed Oct 1, 2025).

43. Shionogi. FETROJAⓇ (cefiderocol) Approved by TFDA in Taiwan. Feb 20, 2024. https://www.shionogi.com/global/en/news/2024/02/2024_0222_2.html (accessed Oct 1, 2025).

44. Shionogi. Regarding the Acquisition of Manufacturing and Marketing Approval for the New Siderophore Cephalosporin Antibiotic FetrojaⓇ(cefiderocol) Intravenous Infusion 1g vial in Japan. Shionogi 2023. Nov 20, 2023. https://www.shionogi.com/global/en/news/2023/11/20231130.html (accessed Oct 1, 2025).

45. Shionogi. Approval for “Fetroja®” (cefiderocol) in South Korea. Shionogi Inc. - a Discovery-Based Pharmaceutical Company; 2025. Feb 28, 2025. https://www.shionogi.com/us/en/news/2025/02/approval-for-fetroja-cefiderocol-in-south-korea.html (accessed Oct 1, 2025).

46. Access To Medicine Foundation. Antimicrobial Resistance Benchmark Opportunities: progress update. Nov, 2023. https://accesstomedicinefoundation.org/medialibrary/231121-amr-benchmark-opportunities-progress-update-november-2023-1700568429.pdf (accessed Oct 1, 2025).

47. Rex JH. Manufacturing underpins access in LMICs: An update on cefiderocol. AMR Solutions; April 14, 2025. <https://amr.solutions/2025/04/14/manufacturing-underpins-access-in-lmics-an-update-on-cefiderocol/> (accessed Oct 1, 2025).

48. Dall C. Agreement aims to expand antibiotic access in poor countries. CIDRAP News; Apr 14, 2023. <https://www.cidrap.umn.edu/agreement-aims-expand-antibiotic-access-poor-countries> (accessed Oct 1, 2025).

49. Global Antibiotic Research & Development Partnership (GARDP). Shionogi, GARDP and CHAI announce landmark license and collaboration agreements to treat bacterial infections by expanding access to cefiderocol in 135 countries. Jun 15, 2022. https://gardp.org/shionogi-gardp-and-chai-announce-landmark-license-and-collaboration-agreements-to-treat-bacterial-infections-by-expanding-access-to-cefiderocol-in-135-countries/ (accessed Oct 1, 2025).

50. Global Antibiotic Research and Development Partnership (GARDP). License agreement overview: Cefiderocol. 2022. <https://gardp.org/wp-content/uploads/2022/10/License-Agreement-Overview-Cefiderocol.pdf> (accessed Oct 1, 2025).

51. Simner PJ, Patel R. Cefiderocol antimicrobial susceptibility testing considerations: the Achilles' heel of the Trojan horse? *Journal of clinical microbiology* 2020; **59**(1): 10.1128/jcm. 00951-20.

52. Viale P, Sandrock CE, Ramirez P, Rossolini GM, Lodise TP. Treatment of critically ill patients with cefiderocol for infections caused by multidrug-resistant pathogens: review of the evidence. *Annals of intensive care* 2023; **13**(1): 52.

53. Rathod D, Chen Z, Slover CM, Kung F, Morita J, Nguyen ST. Cefiderocol For Injection: Compatibility Testing Using the MINI-BAG Plus Container System and the VIAL-MATE Adaptor. *Journal of Pharmacy Technology* 2023; **39**(4): 159-63.

54. Warecki BA, Tomatis PE, Mojica MF, et al. Cefiderocol “under siege”? Understanding the rise of NDM-mediated resistance to novel agents. *Chemical Science* 2025.
